# Electric Fields Can Assist Prebiotic Reactivity on Hydrogen Cyanide Surfaces

**DOI:** 10.1021/acscentsci.5c01497

**Published:** 2026-01-14

**Authors:** Marco Cappelletti, Hilda Sandström, Martin Rahm

**Affiliations:** Department of Chemistry and Chemical Engineering, 11248 Chalmers University of Technology, Gothenburg 412 96, Sweden

## Abstract

Hydrogen cyanide (HCN) is present in many astrochemical environments, including interstellar clouds and comets. On Saturn’s moon Titan, large amounts of HCN ice are present in the atmosphere and, following surface deposition, may influence both chemical and geological evolution. However, despite HCN’s relevance to origin of life chemistry, the physiochemical properties of its solid state remain poorly characterized. For example, the crystals of HCN exhibit a range of rare properties, including pyroelectricity, and the ability to glow and jump under certain conditions. Here we use quantum chemical methods to predict HCN crystal surface energies, from which we derive the needle-like, high-aspect-ratio morphology of HCN nanocrystals. The predicted tips expose high-energy polar facets imbued with strong electric fields. We suggest that the combination of tips of opposite polarity helps to explain the cobweb-structure of solid HCN, and that fracture can transiently expose energetic surfaces, capable of catalysis at low temperature. One such process is predicted to be the near-barrierless formation of isocyanide (HNC) on HCN crystals, following proton addition or abstraction, for example, via radiation or acid/base-chemistry. Such field-assisted surface mechanisms may contribute to HCN-to-HNC isomerization under relevant conditions, and are suggested to explain part of the out-of-equilibrium abundance of HNC in cold environments such as Titan’s atmosphere, and, potentially, in cometary comae.

## Graphical abstract

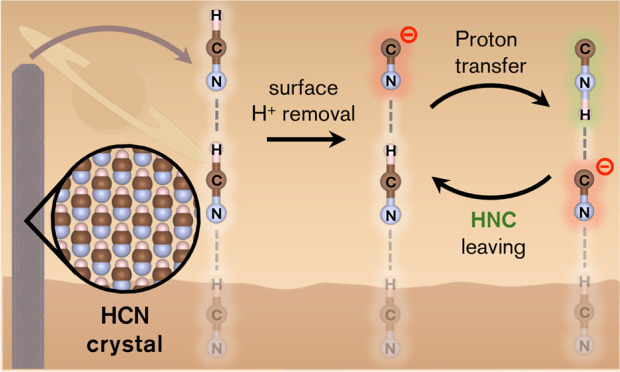

## Introduction

In this work, we calculate the approximate morphology of hydrogen cyanide (HCN) nanocrystals and begin to address the astrochemical consequences of large electric fields generated on the surface of such crystals, including for isomerization of HCN to hydrogen isocyanide (HNC).

HCN is important for many reasons: Aside from its utility as an industrial chemical, its abundance makes it of interest in planetary science, in astrochemistry and as a plausible key building block for the chemistry that preceded the origin(s) of life. HCN has been observed in gas phase in the interstellar medium (ISM) and protostellar environments,^1–4^ in the coma of comets,^5–8^ on carbonaceous chondrites,^9^ and in the atmosphere of various planets,^10,11^ dwarf planets,^12^ and moons.^13–15^ The likelihood of HCN’s role in origin of life chemistry derives from this abundance as well as its established ability to react under various conditions,^16^ yielding complex polymers, and a plentitude of prebiotically relevant molecules, including amino acids and nucleobases.^17–19^

With a melting point of 259 K at 1 atm, HCN can also persist in the solid state in various environments. For example, pockets of solid HCN may be present within water ices in planet-forming regions,^20^ where it could play a role in chemical evolution. While HCN ice has yet to be confirmed in comets, it has been tentatively detected in the atmosphere of Neptune’s moon Triton,^21^ and observed in large amounts in the atmosphere of Saturn’s moon Titan,^22^ an environment we focus on.

HCN is one of the major products of Titan’s upper atmospheric chemistry.^23,24^ After formation, it drifts downward and condenses together with other reaction products into aerosols that feed Titan’s complex yellow haze. Condensation of HCN ice can occur at altitudes below approximately 300 km,^22^ depending on temperature.^25^ At the Huygens site, modeling indicates that HCN begins to condense near ∼75 km, with pure HCN cloud particles extending to ∼30 km.^26^ A south-polar cloud incorporating crystalline HCN was observed at ∼300 km in 2012, later descending toward ∼200 km as the season progressed, implying local temperatures near the HCN frost point (∼125 K) at those altitudes.^27^ Over time, an estimated 2 mm per Myr of HCN ice (or HCN-equivalences in reaction products) is believed to accumulate on Titan’s ∼94 K surface.^28,29^ Deciphering the structure, distribution and properties of HCN ice on Titan is therefore essential for understanding both chemical and geological evolution of this world.^24^ There is growing evidence that ethane and methane, the main components of Titan’s lakes and seas, can intercalate into the HCN crystal lattice, forming cocrystals that could take the role of cryogenic minerals.^30^ Whether solid-state HCN is chemically active in this setting – and to what degree its higher energy isomer HNC can form from it – remains an open question that we will return to discuss.

HCN is one of the most polar naturally occurring molecules, and sports a dipole moment of 2.99 D.^31^ In the liquid state, its dielectric constant is 144.8 at 278 K, notably larger than that of water (85.8 at 278 K).^32,33^ The strong polarity and the ability of HCN to both accept and donate hydrogen bonds ensures a preference for linear chain formation, which leads to strong cooperativity effects.^34–39^ For example, the already sizable dipole moment of HCN increases by 10% in the dimer,^40^ by 20% in the trimer,^41^ and by 40% in a linear chain of 10 molecules.^36^ This effect translates into large oriented electric fields in the crystalline state,^42,43^ and as we shall see, on polar surfaces of the crystal.

The solid-state structure of HCN has been studied with X-ray diffraction,^44^ Raman spectroscopy,^45,46^ and theoretical calculations,^47–50^ revealing two polar crystal phases. The orthorhombic *Imm*2 phase of HCN shown in Figure 1 is the established ground state below 170 K and at atmospheric pressure. At higher temperatures, HCN undergoes a ferroelastic phase transition to a tetragonal *I*4*mm* form.^44,51^

**Figure 1:**
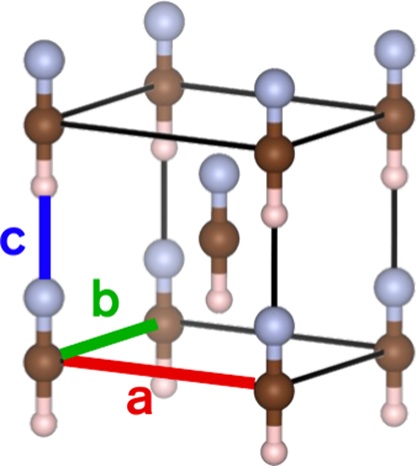
Orthorhombic *Imm*2 phase of the HCN crystal. A permanent electric field is present in the direction of hydrogen-bonded chains of molecules (*c*-axis). The two orthogonal (*a*- and *b*-axis) directions are nonpolar. Hydrogen, carbon and nitrogen atoms are depicted in white-pink, brown, and light blue, respectively.

The nonisotropic nature endows the HCN crystal with interesting properties, including strong pyroelectricity,^44^ and gives rise to peculiar phenomena observed in HCN crystallization experiments, such as electrical breakdowns that are possibly due to electrical charge separations following brittle fracture, and glow.^52,53^ Furthermore, HCN crystals grow into high-aspect ratio needles, which are connected by each other in a “cobweb” framework (Figure 2).

**Figure 2:**
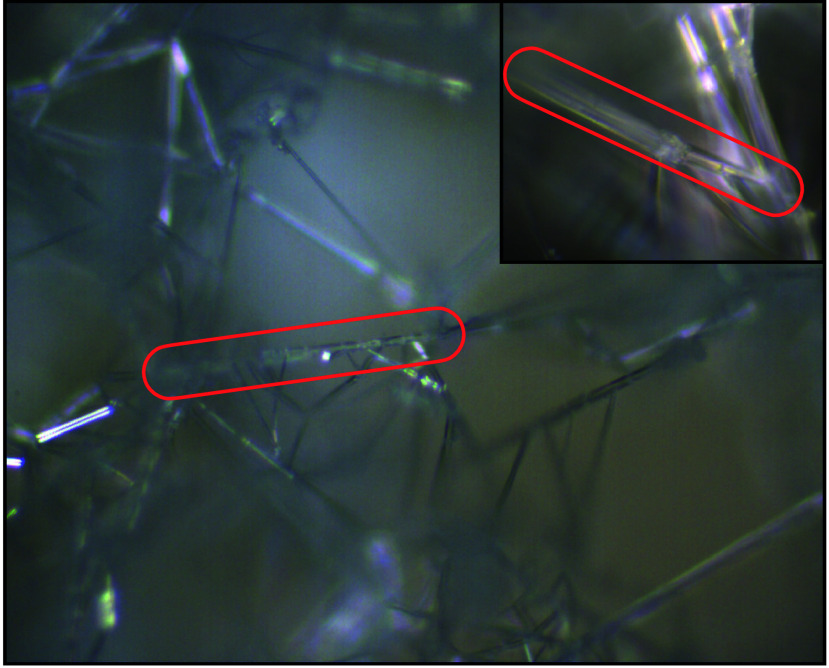
Microscope image of HCN crystals with 10× (full image) and 50× (top right inset) magnification. HCN crystallizes to high aspect-ratio needles (two are highlighted in red), which form a cobweb connectivity. Image reproduced with permission from Morgan L. Cable, Tuan Vu, and Robert Hodyss Courtesy of NASA/JPL-Caltech.

This work is motivated by the potential for large, potentially chemistry-inducing, electric fields on the surfaces of HCN crystals. Electric fields are well established to facilitate chemical reactions and selectivity,^54–56^ and can be mediated by, e.g., enzyme active sites,^57–60^ designed catalysts,^61^ and nanoscale break junctions.^62,63^ However, mainly due to HCN toxicity, the solid-state physiochemical properties of HCN remain understudied. Furthermore, while surface catalysis has been extensively investigated in astrochemistry, the role of surface electric fields is often overlooked.

Of particular interest to us is evaluating an alternative route for HCN ↔ HNC isomerization through surface catalysis. Which processes enable HCN ↔ HNC isomerization remains an open question in astrochemistry. The unimolecular gas-phase isomerization is known to be strongly hindered, both kinetically by a substantial activation energy of ∼200 kJ/mol, and thermodynamically by a positive reaction energy of ∼60 kJ/mol.^64^ Despite these unfavorable energetics, the HNC isomer is frequently found in out-of-equilibrium abundances in various astrochemical environments, where HNC/HCN ratios can range from 0.01 up to 4.5.^1,65–70^ For example, in comets it varies between 0.01 and 0.2, and increases with decreasing heliocentric distance,^65,71–76^ suggesting an in situ production^6,70,74^ that may be enhanced by solar photon flux.^77^ In the higher atmosphere of Titan estimates of the HNC/HCN ratio go as high as 0.3.^78^

The driving forces for HNC formation may in principle be several, including photodissociation of HCN,^74,79^ the degradation of HCN-based polymers or other organics,^74,76^ and various chemical reactions that bypass the neutral isomerization barrier.^6,80–82^ In the solid state, the presence of water ice has been computationally shown to lower the isomerization barrier somewhat, however in such a case HNC formation still remains significantly thermodynamically disfavored by ∼42–44 kJ/mol.^83,84^ Protonation is known to reduce the isomerization barrier to HNC substantially,^85–88^ and protons can be donated by various ions such as H_2_O^+^, or H_3_O^+^,^79^ or by ionization from cosmic radiation.^89^ On Titan specifically, photochemical models indicate that most HNC forms in the ionosphere,^90^ with one important source being HCNH^+^,^28,29,91^ which forms through protonation of HCN. Cosmic rays also penetrate Titan’s dense atmosphere and peaks in ionization at ∼65 km,^92^ where it can drive chemical transformations.

Besides the unexplained high HNC/HCN ratio in Titan’s atmosphere, a second potential anomaly is the sharp decline of HNC with altitude.^93^ Although HNC → HCN is thermodynamically favored, known neutral reisomerization barriers are prohibitively high at Titan temperatures, making chemical reactivity, cosmic rays or photolysis more likely explanations.

In what follows, we predict energies of HCN surfaces, and show them to be commensurate with observed crystal morphology. We also propose a family of novel HCN ↔ HNC isomerization mechanisms, enabled by the ionization of solid HCN polar surfaces, which may contribute to explaining the HNC abundance anomaly in cold HCN-rich environments, such as Titan.

## Results and Discussion

### HCN Surface Energies and Crystal Morphology

The potential for an HCN crystal to drive chemical reactions arguably depends on the fractional area occupied by polar surfaces, where electric fields are strongest. Estimating the prevalence of such surfaces reduces to the question of estimating the shape of HCN nanocrystals. However, as far as we can determine, the equilibrium crystal shape of pure HCN has not been reported, experimentally or computationally. To do so, we rely on Wulff’s theorem, which states that the equilibrium shape of a single crystal, the so-called Wulff construction, is that which minimizes the total surface energy.^94,95^ Surface energies (γ) are here calculated by means of periodic Density Functional Theory (DFT), the details of which are outlined in the Methods section. Because our focus is on cryogenic environments, we limit our study to the orthorhombic *Imm*2 phase. Given that the high-temperature tetragonal *I*4*mm* phase retains the same polar chain topology, we expect our qualitative conclusions to carry over to the 259 K melting point, although quantitative surface energies will be phase dependent.

Although Wulff constructions are exact only in the limit of an infinite number of surface energies, a subset often suffices to determine the approximate crystal morphology. Organic crystals typically obey predictions of Wulff construction quite well.^96^ In this work, we limit ourselves to the surfaces defined by the set of Miller indices (*hkl*) such that |*h*| + |*k*| + |*l*| ≤ 3, plus three additional surfaces which sum equals 4. As we shall see, this selection of surface planes, a subset of which are shown in Figure 3a, suffice for a fair prediction of the overall nanocrystal shape.

**Figure 3:**
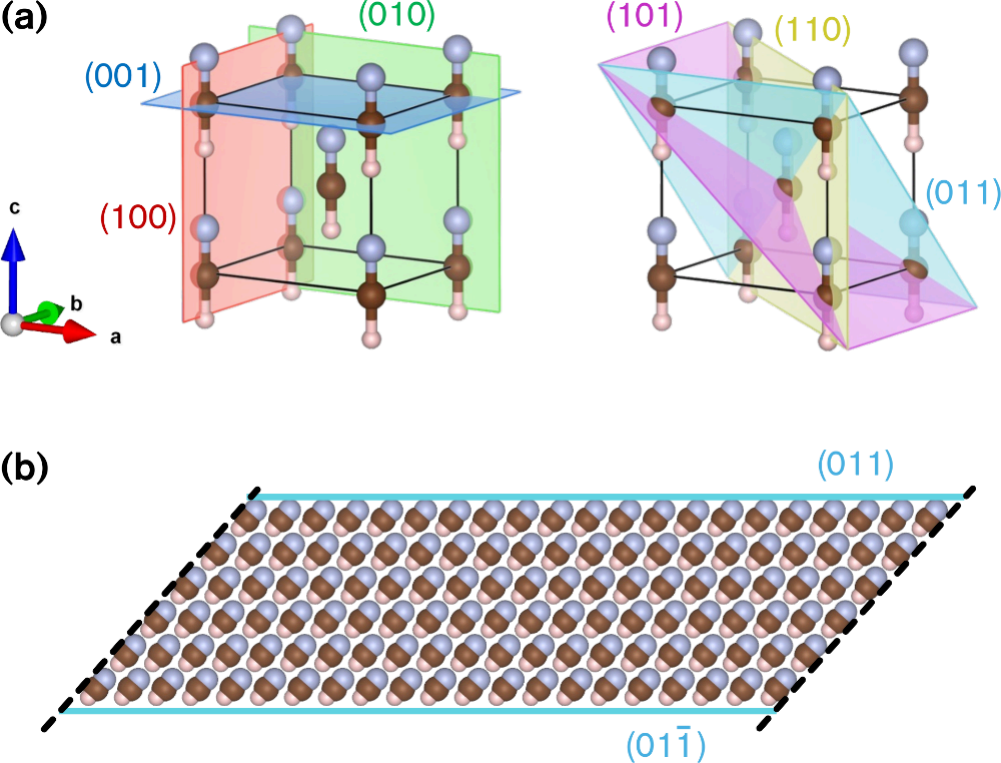
(a) Examples of HCN *Imm*2 crystal lattice planes. (b) Example of computational slab model (here 6 HCN-unit thick) for the (011)/(011̅) surfaces. Slabs of increasing thickness (up to 20 molecular layers) were used to extrapolate the surface energies at infinite thickness (see the Methods section). Nitrogen atoms are depicted in light-blue, carbon atoms in brown, and hydrogen atoms in white-pink.

Figure 3b shows one example of a computational slab model that we have used to estimate surface energies (see the Methods section). In this approach, we cut the bulk HCN crystal along two parallel Miller planes, without breaking any molecular bond. Our computed surface energies thus represent the mean between the two opposite facets of such slabs. This approach is an approximation in the case of polar surfaces, where the asymmetry of the HCN crystal causes the opposite facets to have different terminations (an N-end and an H-end, see Figure 3b), and hence different energies. While we do not expect a substantial discrepancy in energy between the two kinds of terminations, we note that this approach will bias our crystal morphology predictions to identical shapes of N- and H- terminations of HCN crystals. However, pursuing an even more exact model is outside the scope of this work.

Our calculated surface energies, shown in , can be separated into two distinct groups: (i) high energy, polar and (ii) low-energy, nonpolar. The two groups differ in terms of surface energy by an order of magnitude. Most energetic is the {001} surface with a predicted energy of 1.65 J/m^2^, while lowest is {110} at only 0.066 J/m^2^. The energy of a given surface clearly correlates with the amount of N- and H-end termination present on it. We therefore expect the range of all surface energies to be bound by these extrema, i.e., 0.066 ≤ γ ≤ 1.65 J/m^2^.

The surface energies we computed translate into a clear prediction for the morphology of HCN nanocluster as needles (Figure 4a), as expected. To refine our morphology prediction, we have included additional surface energies, estimated through interpolation of the data in Table S2. These additions encompass all surfaces with Miller indexes such that |*h*| + |*k*| + |*l*| ≤ 20 (see the Methods section and Section S2.2). Our resulting best estimate of the single crystal, a cut of which is shown in Figure 4c, features a rounder base compared to Figure 4b, and sharper – pyramid-like – tips. While nonpolar surfaces represent a majority of the surface area, polar surfaces, mainly highly slanted such as {991} and {892}, are exposed to a sizable extent (7%) (Table S3). The latter surfaces are characterized by the presence of steps made of molecules that are likely chemically activated (Figure S5), a possibility that will be studied in future work.

**Figure 4:**
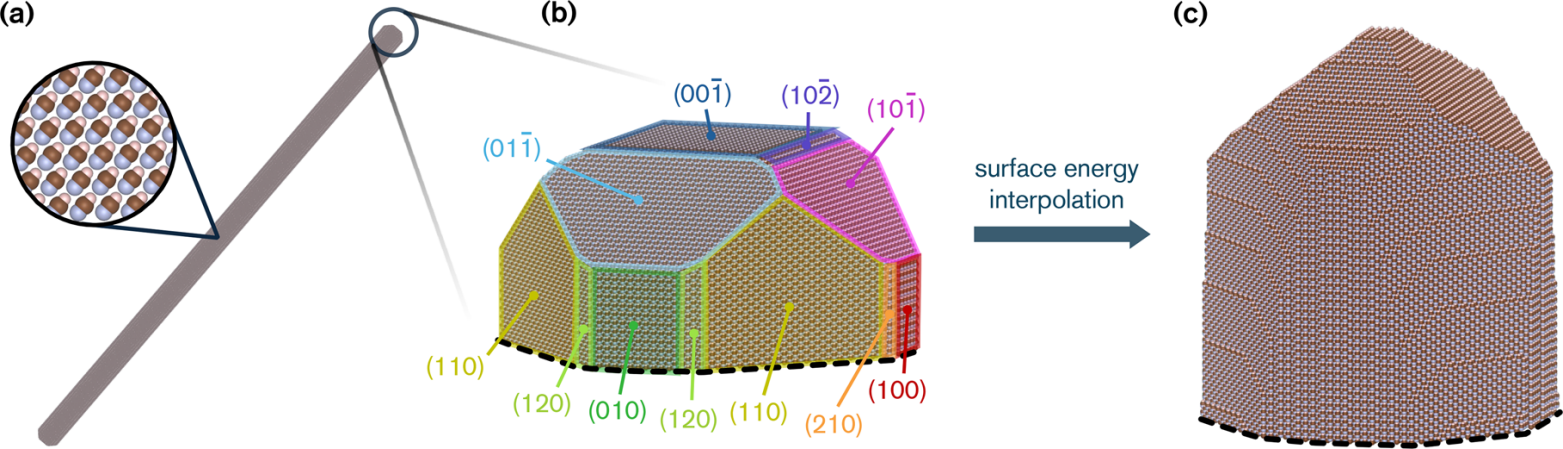
(a) An HCN nanocrystal predicted using Wulff construction and the surface energies of Table S2. The crystal has a length of ∼450 nm, is composed of 10^7^ atoms, and has an aspect ratio of 1:26. (b) Approximate topology of a crystal tip, with Miller lattice planes highlighted with the same color as in Figure 3. (c) Refined topology derived from interpolation of explicitly calculated surface energies (|*h*| + |*k*| + |*l*| ≤ 20, see the Supporting Information).

Our model is of a single HCN crystal in isolation. Of course, the shape of such a crystal may be markedly different in a real chemical environment, where stabilization mechanisms, such as surface reconstruction and charge redistribution, may occur.^97–99^ In astrochemical environments, chemisorption of various molecules and photoexcitation processes may also take place due to exposure to ionizing particles and high-energy photons.^89,100^ However, our model do agree with several observations of HCN beside the needle shape. For example, we have tested how sensitive our prediction is to size of the crystals, and found that their aspect ratio does not vary substantially, and converges to ca. 1:26 (Figure S3), seemingly well in line with macroscopic HCN crystals (Figure 2). The predicted high energy of the crystal tips also offers an explanation for the experimentally observed “cobweb” features, in which no tips appear exposed (Figure 2), but instead act as a nexus for several crystals to join. It is likely that the electrostatic fields exerted at the tips, favor the linking of opposite polar ends, thus preventing the formation of exposed polar interfaces.

### The Intrinsic Electric Fields of HCN

The magnitude of the electrostatic potential generated at polar interfaces of solid HCN provides one measure of its potential catalytic properties. To estimate an upper bound for the electric field present at such polar surfaces, we employ a model composed of a single, isolated chain of HCN molecules, effectively representing the behavior of the {001} surface. The electric field shown in Figure S6 is calculated at 1.9 Å from the edge of such a chain, which is the distance of a typical hydrogen bond. In this way, we estimate the electric field that a molecule adsorbed on the polar surface would experience.

As expected from prior work,^36,39^ the electric field we compute increases with the number of linearly coordinated HCN and converges to a value of 1.10 V/Å at the H-end, and of 1.25 V/Å at the N-end. These electric fields correspond to a ∼20% increase relative to the isolated HCN molecule (0.92 and 1.01 V/Å), and is evidence for a large cooperative effect, in line with previous predictions.^34,37,38^

We motivate the use of a chain model by the higher level of theory it permits compared to periodic slab models of the {001} surface (Figure S7). Slab models appear to predict slightly lower field strengths (e.g., 1.20 → 0.95 V/Å for an H-terminated surface, Figure S7) compared to a single chain-model but suffer noise and method sensitivity. We will return to analyze the electronic structure of HCN crystal surfaces responsible for such differences in future work.

Regardless of the model choice, the predicted electric fields on polar surfaces are large. Linear association of HCN is clearly able to generate field strengths of the same order of magnitude as proteins and scanning tunneling microscopy tips (Figure S6), both known to facilitate chemical transformations.^101–103^ For example, a field of ∼0.9 V/Å is able to induce the dissociation of weakly bonded molecules, e.g., in Na_2_, Li_2_.^56^

We think this insight into electrostatics and structure can be of particular importance for understanding processes in cryogenic environments in the Solar System and beyond, where thermally hindered reaction pathways might proceed with the help of electric-field enabled surface catalysis.

### Surface Mechanisms for HCN ↔ HNC Isomerization

#### Cationic Surface Catalysis

Strong electric fields present at polar HCN surfaces are expected to influence adsorbates and to modify the properties of the outermost HCN. We have predicted the proton affinity of surface HCN by means of a linear chain model at 90 K, the temperature at the surface of Titan, and at 259 K, near the melting point of HCN (see the Methods section). The electrostatic cooperative effects are sufficiently pronounced to increase the proton affinity of the HCN cluster by approximately 20% compared to the HCN molecule (Table S4). Our predicted value of 845 kJ/mol at 90 K and 850 kJ/mol at 259 K is nearly identical to that of gas-phase ammonia (854 kJ/mol at 298 K),^104^ a strong Brønsted base. In this protonation process, the formed HCNH^+^ cation is not a local minimum: instead, a barrier-less proton transfer occurs, effectively relocating the cation one monolayer deeper into the crystal. As illustrated in the left panel of Figure 5, this process yields a new surface species: the aforementioned isomer of HCN, hydrogen isocyanide (HNC). Notably, the same isomerization process could happen if HCNH^+^ is adsorbed directly onto the HCN surface from the environment, as might happen in Titan’s atmosphere.

**Figure 5:**
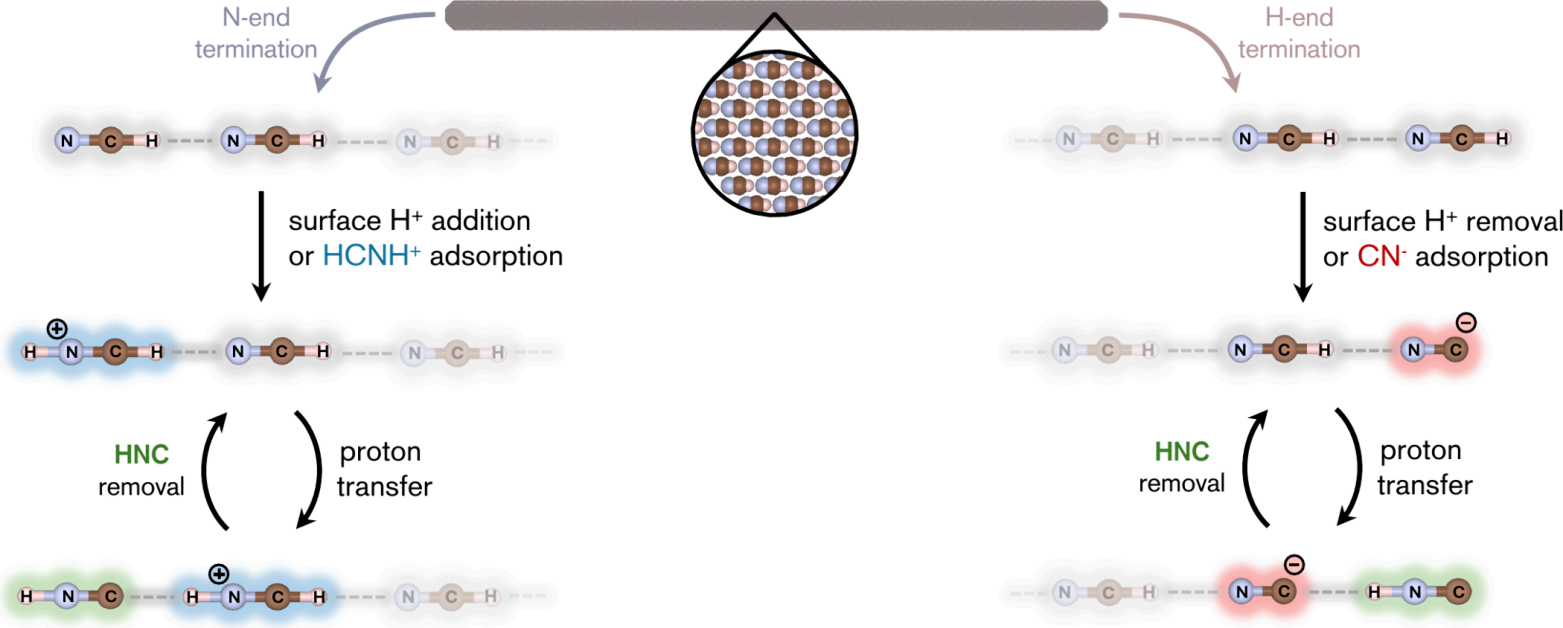
HCN-to-HNC isomerization can be catalyzed by exposed high energy HCN crystal surfaces. Left scheme: Protonation of an N-terminated surface leads to a terminal HCNH^+^ (light blue), and subsequent barrierless proton transfer to generate a terminal HNC (green). Solar wind and galactic cosmic rays are examples of sources of protons. Right scheme: Proton removal (by, e.g., ionizing radiation or acid–base chemistry) of an H-terminated surface generates a terminal CN^–^ (red), which spontaneously migrate into the crystal via a proton transfer reaction that creates a terminal HNC molecule (green). Following sublimation or dissolution of the formed terminal HNC, both processes can continue, ablating the surface.

It is notable that the HCN surface not only catalyzes HNC formation, but also switches the thermodynamic preference relative to the noncatalyzed the gas-phase mechanism: whereas gas-phase proton transfer between HCNH^+^ and HNC to yield HCN and HCNH^+^ is barrierless and exergonic, HNC formation becomes favored on the protonated surface once the chain is composed of four or more units (Figure S8). This result is in line with previous computational studies focused on ionized (open-shell) clusters,^86^ and, in our calculations, the energetics of the process correlates with the intrinsic surface electric field (Figure S8). We therefore assert that such surface-catalyzed HCN ↔ HNC isomerization is electrostatically induced.

Following the formation of a terminal HNC, one can imagine a subsequent step in which the HNC product dissociates from the surface, either via sublimation or solvation into a surrounding chemical environment, thereby regenerating HCNH^+^ at the surface. This cation can again undergo thermodynamically spontaneous proton transfer into the crystal, propagating a surface-catalyzed isomerization cycle (left panel of Figure 5). The rate-determining step in this mechanism is the dissociation of HNC, for which we estimate a Gibbs energy barrier of approximately 75, 62, and 52 kJ/mol at 90, 180, and 259 K, respectively (Figure S10). In other words, while this barrier is far too large to allow for thermal desorption of HNC on the surface of Titan, it does permit reactions on the time scale of days at ∼180 K, close to the temperature where HCN ice is believed to form and persist on Titan. At higher temperatures near the melting point (259 K), the barrier is low enough to allow isomerization on the time scale of milliseconds. These estimates were obtained using the Eyring equation, assuming first-order reaction kinetics.

#### Anionic Surface Catalysis

The larger crystal-induced dipole moment also stabilizes the formation of the cyanide anion. Terminal HCN molecules are predicted to exhibit a ∼10% lower gas-phase acidity (GA) than isolated HCN, with a predicted GA of 1310 kJ/mol at 259 K. Since lower GA values indicate higher acidity, this implies that terminal HCN is more acidic, surpassing than that of HBr (1331 kJ/mol at 298.15 K)^105^ (see section S3.2). The deprotonation could, for example, be achieved by irradiation. Alternatively, a cyanide anion, found in Titan upper atmosphere,^106,107^ may be adsorbed to the H-terminated surface. In such cases, the resulting cyanide anion is predicted to exist as a metastable transient on the surface. This anion can rapidly migrate into the crystal via proton transfer from an underlying HCN molecule, in an exoergic process with a calculated reaction Gibbs energy of ∼ −5 kJ/mol at 90 K. We compute the reaction energy barrier for this step to be very small, ∼4 kJ/mol, which corresponds to a first order reaction time scale in the order of picoseconds at 90 K. Similarly to the cationic mechanism, this process appears to be electrostatically induced, as both the reaction energy and the reaction barrier linearly correlate with the electric field at the H-end of the chain (Figure S9).

The right panel of Figure 5 illustrates how this alternative pathway, initiated by deprotonation or surface association of CN^–^, and followed by proton transfer, constitutes a second mechanism for surface-catalyzed HCN ↔ HNC isomerization. As in the cationic mechanism, the rate-determining step is the desorption of the HNC product from the surface (estimated between 52 and 75 kJ/mol, depending on the temperature, c.f., Figure S10). Also similar to the cationic case, the anionic mechanism represents a fundamentally different situation compared to when uncatalyzed in the gas-phase: the reversed proton transfer from HNC to CN^–^, forming CN^–^ and HCN, is exergonic and barrierless. These examples are indicative of a remarkable catalytic potential of polar HCN surfaces.

While tunneling may contribute to proton transfer at cryogenic temperatures, we have not explicitly included such effects in our calculations. This omission is motivated by the negligible proton transfer barriers we compute. The overall reaction rate is completely governed by the much larger barrier for HNC desorption, and tunneling is not expected to alter our conclusions.

### Speculation on Astrochemical Relevance

We think that the mechanisms outlined in Figure 5 offer a plausible explanation for the observed abundance anomaly of HNC in Titan’s atmosphere, and for how solid-phase HCN may participate in dynamic, surface-driven transformations at low temperatures. While these mechanisms are but models, they highlight how surface-catalyzed chemistry may be particularly relevant in Titan’s complex chemical environment. HCN is one of the major products of the atmospheric photochemistry of Titan,^23,24^ and micron-sized HCN ice particles have been observed at high altitude near its southern pole.^22^ This environment is bathed in ionizing radiation from the Sun and Saturn’s magnetosphere, and the atmospheric layers below are rich in photochemically generated ions, including HCNH^+^,^29^ that could facilitate HCN → HNC isomerization via the mechanisms we have described.

Once formed, HNC can remain bound to the surface or be released into the gas phase, either thermally when the particles are lofted into warmer atmospheric layers, or when they are exposed to UV photons or charged particle irradiation. While our Gibbs energy estimates suggest that temperatures around 180 K suffice for quantitative desorption on time scales of days, we speculate that nonthermal processes may accelerate such release and become dominant at lower temperatures. Galactic cosmic rays and their secondary particles also penetrates Titan’s dense atmosphere, providing an additional energy source that can further process aerosol and ice surfaces over long time scales.^92^ Modeling such processes lies beyond the scope of our work, but we can provide a simple estimate: solar UV photons with wavelengths greater than 220 nm penetrate deep into Titan’s atmosphere, with photon fluxes of ∼10^10^ and ∼10^7^ photons cm^–2^ nm^–1^ s^–1^ at ∼ 200 and ∼75 km, respectively.^29^ Given typical values reported for molecular ices,^108–111^ we adopt a representative photodesorption yield of 10^–3^ molecules per incident photon. If we further assume an effective spectral bandwidth of 50 nm, a polar surface exposure of 7%, and a 10% HNC surface coverage, we obtain desorption rates (per unit of total HCN crystal surface area) of order ∼10^6^ and ∼10^3^ molecules cm^–2^ s^–1^ at ∼200 and ∼75 km, respectively. These estimates suggests that UV-induced desorption pathways may occur on time scales of years at ∼200 km, but not closer to the surface.

Following HNC desorption, new HNC is regenerated that could either become trapped in the lattice or continue the release. This process would provide a dynamic route for modulating the gas-phase nitrile composition without requiring bulk sublimation of HCN ice. It could also contribute to the vertical redistribution and chemical evolution of nitriles in Titan’s stratified atmosphere.

In the case of comets, observations have consistently shown that the gas-phase HNC/HCN ratio increases as comets approach the Sun.^65,71–75^ This has been interpreted as evidence of in situ chemical production of HNC, rather than it being a parent molecule.^6,70,112^ Our computed binding energy for HNC on HCN crystal surfaces (∼75 kJ/mol) suggests that at low temperatures, HNC formed via surface-catalyzed isomerization would remain chemisorbed. However, near perihelion, solar heating can supply sufficient energy to desorb HNC from the outermost surface layer and release it into the coma. In addition to thermal desorption, nonthermal processes such as UV photodesorption and solar-wind sputtering should also be enhanced near perihelion. In this scenario, HCN crystals, if present, could act as catalytic reservoirs: storing HCN in the solid phase, converting a fraction into HNC at their polar surfaces, while releasing and producing more HNC when heated. Such processes align with observations of increased HNC production in comets at small heliocentric distances, where thermal and radiative processing of near-surface ices is most intense.^113^

Titan and comets are two distinct examples where nontrivial surface chemistry involving HCN, and likely HNC, can occur. Both settings are characterized by low temperatures, exposure to variable thermal and radiative conditions, and observed signatures of HNC formation under circumstances where purely gas-phase or higher-temperature explanations appear insufficient. While solid HCN has been directly observed on Titan, its presence in comets remains inferred rather than confirmed. Nevertheless, if HCN condenses in cometary ices, the catalytic mechanisms proposed here may similarly operate to modulate coma composition. These processes point to a broader role for surface-mediated HCN reactivity in low-temperature environments. The discussed mechanisms might not only explain specific compositional anomalies, but also enable otherwise inaccessible reaction pathways, such as the formation of more complex nitrile derivatives or prebiotic precursors, in cold regions of the Solar System and beyond.

## Conclusions

In this work, we computationally investigate the catalytic potential of solid hydrogen cyanide (HCN) surfaces. Solid HCN is either expected or confirmed to be present in a variety of astrochemical environments, and we focus our analysis on cryogenic settings, with a particular emphasis on the atmosphere and surface of Saturn’s moon Titan.

Our calculations show that HCN surface energies vary by more than an order of magnitude, explaining the growth of highly elongated, needle-like crystals. This anisotropy in surface stability helps explain experimentally observed “cobweb” morphologies, which may arise from electrostatic attraction between oppositely polarized, N- and H-terminated crystal facets. The polar surfaces, being significantly higher in energy, also exhibit strong oriented electric fields. These fields originate from the cooperative alignment of molecular dipoles and reach magnitudes capable of significantly perturbing local electrostatic environments and surface-bound chemistry.

We identify two classes of surface-facilitated mechanisms that enable the isomerization of HCN to its higher-energy isomer HNC. In the first, protonation of a terminal HCN unit triggers a barrierless or low-barrier rearrangement to HNC, followed by proton transfer into the crystal. In the second, surface deprotonation generates a transient cyanide anion that undergoes a similar proton transfer cascade. In both cases, HNC forms only at the surface, and its removal – whether by thermal desorption or radiative processing – acts as the rate-determining step for continued isomerization. These mechanisms offer a plausible explanation for the observed anomalously high abundance of HNC in environments such as Titan’s atmosphere and, potentially, in cometary comae.

Validation of our predictions would benefit from laboratory studies of HCN surface chemistry under cryogenic conditions. One particularly relevant experiment would be to test whether physical stimuli, such as crushing HCN crystals in the presence of reagents like water, can expose high-energy surfaces and thereby accelerate prebiotically relevant chemical transformations. Observational efforts targeting HNC/HCN ratios across environments and temperatures could further constrain the relevance and prevalence of these mechanisms under astrophysical conditions.

## Methods

### Periodic Calculations

Calculations on the HCN crystal and on all slab models were performed with the Vienna ab initio simulation package^114^ (VASP) version 6.4.1, using standard projected augmented wave (PAW) potentials.^115^ The Perdew–Burke–Ernzerhof (PBE) exchange-correlation functional was used combined with the D3­(BJ) dispersion correction.^116^ A planewave energy cutoff of 900 eV was used throughout. All calculations relied on Γ-centered *k*-point meshes: 5 × 5 × 5 for the bulk crystal, and 5 × 5 × 1, 5 × 1 × 5, or 1 × 5 × 5 for slab models periodic in the *xy*, *xz*, or *yz*, directions, respectively. The energy cutoff and the *k*-point density were converged to below 0.1 meV/atom. Calculations on polar slabs included the dipole moment correction to the potential and forces as implemented in VASP and described in reference 117. Validation of the computational setup against experimental lattice parameters is provided in the Supporting Information (Table S1).

### Surface Energy

Surface slabs were constructed based on the PBE-D3BJ optimized bulk structure of solid HCN. Each slab exposes two opposing surface terminations (H- and N-), e.g., corresponding to the (001) and (001̅) planes, respectively, with a 40 Å thick vacuum layer. The average surface energy, γ, of these surfaces was calculated as in eq 1:

1 $$\gamma =\frac{1}{2A}\left(E_{slab}-nE_{bulk}\right)$$

where *A* is the surface area of the slab, *E*_slab_ is the total energy of the slab model, *n* is the number of HCN molecules in the slab model and *E*_bulk_ is the energy per HCN molecule in the bulk crystal. The factor of 1/2 accounts for the two surfaces in the slab. Surface energies were computed for slabs with increasing thickness (from 4 to 20 molecular layers), and the values of γ were extrapolated to the limit *n* → *∞* to approximate the thickness of macroscopic crystals (Figure S1). For our best estimate of the crystal shape, additional surface energies were interpolated from the computed data (Section S2.2). Interpolation was performed using radial basis function (Rbf), as implemented in SciPy version 1.15.2.^118^

### HCN Nanocrystal Morphology

Crystal morphology was estimated using the Wulff construction, as implemented in the WulffPack Python package.^119^ This method determines the crystal shape by minimizing the total surface energy, and yields an exact result if the complete set of surface energies is known. To generate the nanoclusters shown in Figure 4, we used the surface energies listed in Table S2 as input. The resulting single-crystal morphology also depends on the number of atoms in the cluster, which is provided as an additional input parameter. Figure S3 shows convergence of morphology with respect to the number of atoms.

### Molecular Cluster Optimization

All molecular cluster optimizations and subsequent electric field analyses were performed with Gaussian16, revision B.01.^120^ Structural relaxation relied on the B3LYP functional, with D3­(BJ) dispersion corrections and the 6-311++G­(d,p) basis set. Final electronic energies were refined using ORCA version 6.0.1,^121^ with the domain-based local pair natural orbital (DLPNO) local correlation approximation to coupled-cluster theory including single, double, and perturbative triple excitations, DNLPO-CCSD­(T),^122^ and the aug-cc-pVTZ basis set, giving *E*_el_^CCSD(*T*)^. Final Gibbs energies, *G*, and enthalpies, *H*, were computed as eq 2 and 3, respectively:

2 $$G=E_{el}^{CCSD\left(T\right)}+\Delta G_{th}\left(T\right)$$

3 $$H=E_{el}^{CCSD\left(T\right)}+\Delta H_{th}\left(T\right)$$

where Δ*G*_th_(*T*) and Δ*H*_th_(*T*) includes thermal, rotational and translational enthalpic and (for *G*) entropic corrections evaluated at the B3LYP-D3­(BJ)/6–311++G­(d,p) level of theory. Small vibrational frequencies (<100 cm^–1^) have been treated according to the rigid-rotor-harmonic-oscillator (RHHO) approximation.^123^

### Proton Affinity and Gas-Phase Acidity

The proton affinity (PA) at 90 K, representative of the surface temperature of Titan, was computed as the negative enthalpy change, −Δ*H*, of the reaction in eq 4:

4 $$M+H^{+}\to {\mathrm{MH}}^{+}$$

The gas-phase acidity (GA) was computed as the Gibbs energy change, Δ*G*, of the reaction in eq 5:

5 $$X^{‐}+H^{+}\to \mathrm{XH}$$

Calculations were also performed at 298.15 K, providing values in good agreement with literature data (see Table S4).

### Electric field

The electrostatic potential from the linear chain model was extracted using the *cubegen* utility in *Gaussian16*, and sampled at points 1.9 Å from the outermost hydrogen atom (hydrogen or nitrogen). The local electric field was then calculated as the gradient of this potential. For the slab model, the local electrostatic potential was extracted from a LOCPOT file generated using a dense fast Fourier transform (FFT) mesh in VASP.

## Supplementary Material





## Notes

HCN lattice parameters and surface energies, Wulff constructions aspect ratio, surface energy interpolation, electric field plot, chemical properties of HCN clusters, dissociation energies of HCN and HNC from HCN clusters (PDF)
Transparent Peer Review report available (PDF)

## #

(H.S.) Department of Chemistry, Technical University of Munich, James-Franck-Str. 1, 85748, Garching b. Munich, Germany

## Abbreviations

DFT: density functional theory
HCN: hydrogen cyanide
HNC: hydrogen isocyanide.

## References

[1] Hirota T., Yamamoto S., Mikami H., Ohishi M. (1998). Abundances of HCN and
HNC in Dark Cloud Cores. Astrophys. J..

[2] Rice T. S., Bergin E. A., Jørgensen J. K., Wampfler S. F. (2018). Exploring the Origins
of Earth’s Nitrogen: Astronomical Observations of Nitrogen-Bearing
Organics in Protostellar Environments. Astrophys.
J..

[3] Jewell P. R., Snyder L. E., Schenewerk M. S. (1986). Detection
of Hydrogen Cyanide Emission
from the Peculiar Oxygen–Rich Evolved Star IRC + 10420. Nature.

[4] Thi W. F., Van Zadelhoff G. J., Van Dishoeck E. F. (2004). Organic Molecules in Protoplanetary
Disks around T Tauri and Herbig Ae Stars. Astron.
Astrophys..

[5] Dello
Russo N., Kawakita H., Vervack R. J., Weaver H. A. (2016). Emerging
Trends and a Comet Taxonomy Based on the Volatile Chemistry Measured
in Thirty Comets with High-Resolution Infrared Spectroscopy between
1997 and 2013. Icarus.

[6] Rodgers S. D., Charnley S. B. (1998). HNC and HCN in Comets. Astrophys.
J..

[7] Wirström E. S., Lerner M. S., Källström P., Levinsson A., Olivefors A., Tegehall E. (2016). HCN Observations of Comets C/2013
R1 (Lovejoy) and C/2014 Q2 (Lovejoy). Astron.
Astrophys..

[8] Huebner W. F., Snyder L. E., Buhl D. (1974). HCN Radio
Emission from Comet Kohoutek
(1973f). Icarus.

[9] Pizzarello S. (2012). Hydrogen Cyanide
in the Murchison Meteorite. Astrophys J. Lett..

[10] Tokunaga A. T., Beck S. C., Geballe T. R., Lacy J. H., Serabyn E. (1981). The Detection
of HCN on Jupiter. Icarus.

[11] Lellouch E., Romani P. N., Rosenqvist J. (1994). The Vertical
Distribution and Origin
of HCN in Neptune’s Atmosphere. Icarus.

[12] Lellouch E., Gurwell M., Butler B., Fouchet T., Lavvas P., Strobel D. F., Sicardy B., Moullet A., Moreno R., Bockelée-Morvan D., Biver N., Young L., Lis D., Stansberry J., Stern A., Weaver H., Young E., Zhu X., Boissier J. (2017). Detection of CO and HCN in Pluto’s Atmosphere
with ALMA. Icarus.

[13] Tanguy L., Bézard B., Marten A., Gautier D., Gérard E., Paubert G., Lecacheux A. (1990). Stratospheric Profile of HCN on Titan
from Millimeter Observations. Icarus.

[14] Peter J. S., Nordheim T. A., Hand K. P. (2024). Detection of HCN and Diverse Redox
Chemistry in the Plume of Enceladus. Nature
Astronomy.

[15] Hanel R., Conrath B., Flasar F. M., Kunde V., Maguire W., Pearl J., Pirraglia J., Samuelson R., Herath L., Allison M., Cruikshank D., Gautier D., Gierasch P., Horn L., Koppany R., Ponnamperuma C. (1981). Infrared Observations of the Saturnian System from
Voyager 1. Science (1979).

[16] Ruiz-Bermejo M., de la Fuente J. L., Pérez-Fernández C., Mateo-Martí E. (2021). A Comprehensive
Review of HCN-Derived Polymers. Processes.

[17] Oró J. (1960). Synthesis
of Adenine from Ammonium Cyanide. Biochem. Biophys.
Res. Commun..

[18] Ferris J. P., Joshi P. C., Edelson E. H., Lawless J. G. (1978). HCN: A
Plausible
Source of Purines, Pyrimidines and Amino Acids on the Primitive Earth. J. Mol. Evol.

[19] Ruiz-Bermejo M., Zorzano M.-P., Osuna-Esteban S. (2013). Simple Organics and Biomonomers Identified
in HCN Polymers: An Overview. Life.

[20] Bergner J. B., Rajappan M., Öberg K. I. (2022). HCN Snow
Lines in Protoplanetary
Disks: Constraints from Ice Desorption Experiments. Astrophys. J..

[21] Burgdorf M., Cruikshank D. P., Dalle Ore C. M., Sekiguchi T., Nakamura R., Orton G., Quirico E., Schmitt B. (2010). A Tentative
Identification of HCN Ice on Triton. Astrophysical
Journal Letters.

[22] De
Kok R. J., Teanby N. A., Maltagliati L., Irwin P. G. J., Vinatier S. (2014). HCN Ice in Titan’s High-Altitude
Southern Polar Cloud. Nature.

[23] Lavvas P. P., Coustenis A., Vardavas I. M. (2008). Coupling Photochemistry with Haze
Formation in Titan’s Atmosphere, Part II: Results and Validation
with Cassini/Huygens Data. Planet Space Sci..

[24] Nixon C. A. (2024). The Composition
and Chemistry of Titan’s Atmosphere. ACS Earth Space Chem..

[25] Fulchignoni M., Ferri F., Angrilli F., Ball A. J., Bar-Nun A., Barucci M. A., Bettanini C., Bianchini G., Borucki W., Colombatti G., Coradini M., Coustenis A., Debei S., Falkner P., Fanti G., Flamini E., Gaborit V., Grard R., Hamelin M., Harri A. M., Hathi B., Jernej I., Leese M. R., Lehto A., Lion Stoppato P. F., López-Moreno J.
J., Mäkinen T., McDonnell J. A. M., McKay C. P., Molina-Cuberos G., Neubauer F. M., Pirronello V., Rodrigo R., Saggin B., Schwingenschuh K., Seiff A., Simões F., Svedhem H., Tokano T., Towner M. C., Trautner R., Withers P., Zarnecki J. C. (2005). In Situ Measurements of the Physical
Characteristics of Titan’s Environment. Nature.

[26] Lavvas P., Griffith C. A., Yelle R. V. (2011). Condensation in Titan’s Atmosphere
at the Huygens Landing Site. Icarus.

[27] Hanson, L. E. ; French, R. S. ; Waugh, D. W. ; Barth, E. L. ; Anderson, C. M. The Evolution of Titan’s Cold South Polar Cloud. Geophys. Res. Lett. 2025, 52 (9). 10.1029/2024GL113415.

[28] Willacy K., Allen M., Yung Y. (2016). A New Astrobiological
Model of the
Atmosphere of Titan. Astrophys. J..

[29] Vuitton V., Yelle R. V., Klippenstein S. J., Hörst S. M., Lavvas P. (2019). Simulating the Density of Organic
Species in the Atmosphere
of Titan with a Coupled Ion-Neutral Photochemical Model. Icarus.

[30] Izquierdo-Ruiz F., Cable M. L., Hodyss R., Vu T. H., Sandström H., Lobato A., Rahm M. (2025). Hydrogen Cyanide and
Hydrocarbons
Mix on Titan. Proc. Natl. Acad. Sci. U.S.A..

[31] Bhattacharya B. N., Gordy W. (1960). Observation of Pi Stark Components in Microwave Spectroscopy: Precision
Measurements on HCN. Phys. Rev..

[32] Coates G. E., Coates J. E. (1944). Studies on Hydrogen
Cyanide. Part XIII. The Dielectric
Constant of Anhydrous Hydrogen Cyanide. Journal
of the Chemical Society (Resumed).

[33] Malmberg C. G., Maryott A. A. (1956). Dielectric Constant of Water from 0° to 100 °C. J. Res. Natl. Bur Stand (1934).

[34] de
Oliveira P. M. M. C., Silva J. A. B., Longo R. L. (2017). Benchmark, DFT Assessments,
Cooperativity, and Energy Decomposition Analysis of the Hydrogen Bonds
in HCN/HNC Oligomeric Complexes. J. Mol. Model.

[35] Sánchez M., Provasi P. F., Aucar G. A., Alkorta I., Elguero J. (2005). Theoretical
Study of HCN and HNC Neutral and Charged Clusters. J. Phys. Chem. B.

[36] Adrian-Scotto M., Vasilescu D. (2007). Density Functional
Theory Study of (HCN)­n Clusters
up to n = 10. Journal of Molecular Structure:
THEOCHEM.

[37] Freitas D. P., Pansini F. N. N., Varandas A. J. C. (2023). Linear
and Cyclic (HCN)­n Clusters:
A DFT Study of IR and Raman Spectra. Chem. Phys.
Lett..

[38] Góra R. W., Zaleśny R., Zawada A., Bartkowiak W., Skwara B., Papadopoulos M. G., Silva D. L. (2011). Large Changes of
Static Electric Properties Induced by Hydrogen Bonding: An Ab Initio
Study of Linear HCN Oligomers. J. Phys. Chem.
A.

[39] Brandão I., Rivelino R., Fonseca T. L., Castro M. A. (2013). An Ab Initio Study
of Electric Properties of Linear (HCN)N and (HNC)N Aggregates in Gas
Phase. Chem. Phys. Lett..

[40] Campbell E. J., Kukolich S. G. (1983). Electric Dipole
Moments of (HC15N)­2 and HC15NH35Cl. Chem. Phys..

[41] Ruoff R. S., Emilsson T., Klots T. D., Chuang C., Gutowsky H. S. (1988). Rotational
Spectrum and Structure of the Linear HCN Trimer. J. Chem. Phys..

[42] Bounds D. G., Hinchliffe A., Munn R. W., Newham R. J. (1974). The Dipole Moment
of HCN in the Crystal. Chem. Phys. Lett..

[43] Panas I. (1993). Theoretical
Molecular Dipole Moment Derivatives in Solid HCN. Chem. Phys. Lett..

[44] Dulmage W. J., Lipscomb W. N. (1951). The Crystal Structures
of Hydrogen Cyanide, HCN. Acta Crystallogr..

[45] Aoki K., Baer B. J., Cynn H. C., Nicol M. (1990). High-Pressure Raman
Study of One-Dimensional Crystals of the Very Polar Molecule Hydrogen
Cyanide. Phys. Rev. B.

[46] Pézolet M., Savoie R. (1969). Raman Spectra of Liquid
and Crystalline HCN and DCN. Can. J. Chem..

[47] Milman V., Winkler B. (1999). Ab Initio Modeling
in Crystallography. International Journal of
Inorganic Materials.

[48] Chall M., Winkler B., Milman V. (1996). Ab Initio Calculation
of the High-Pressure
Behaviour of Hydrogen Cyanide. J. Phys.: Condens.
Matter.

[49] Panas I. (1992). A Self-Consistent
Crystal Field Approach to the Structures of Molecular Crystals Applied
to Solid HCN. Chem. Phys. Lett..

[50] Peng J., Zhang S., Refson K., Dove M. T. (2022). The Ferroelastic
Phase Transition in Hydrogen Cyanide Studied by Density Functional
Theory. J. Phys.: Condens. Matter.

[51] Giauque W. F., Ruehrwein R. A. (1939). The Entropy
of Hydrogen Cyanide. Heat Capacity, Heat
of Vaporization and Vapor Pressure. Hydrogen Bond Polymerization of
the Gas in Chains of Indefinite Length. J. Am.
Chem. Soc..

[52] Mozhaev, P. S. ; Kichigina, G. A. ; Kiryukhin, D. P. Radiation-Induced Polymerization of Hydrogen Cyanide. High Energy Chemistry 1994, 29 (1).

[53] Mozhaev P. S., Kiryukhin D. P., Kichigina G. A., Barkalov I. M. (1994). The Formation of
Molecular Compounds Following Radiolysis of Solid Hydrogen Cyanide. Mendeleev Commun..

[54] Shaik S., Ramanan R., Danovich D., Mandal D. (2018). Structure and Reactivity/Selectivity
Control by Oriented-External Electric Fields. Chem. Soc. Rev..

[55] Shaik S., Danovich D., Joy J., Wang Z., Stuyver T. (2020). Electric-Field
Mediated Chemistry: Uncovering and Exploiting the Potential of (Oriented)
Electric Fields to Exert Chemical Catalysis and Reaction Control. J. Am. Chem. Soc..

[56] Stuyver T., Danovich D., Joy J., Shaik S. (2020). External Electric Field
Effects on Chemical Structure and Reactivity. Wiley Interdiscip Rev. Comput. Mol. Sci..

[57] Li N., Yan S., Wu P., Li J., Wang B. (2024). Local Electric Fields
Drives the Proton-Coupled Electron Transfer within Cytochrome P450
Reductase. ACS Catal..

[58] Stuyver T., Ramanan R., Mallick D., Shaik S. (2020). Oriented (Local) Electric
Fields Drive the Millionfold Enhancement of the H-Abstraction Catalysis
Observed for Synthetic Metalloenzyme Analogues. Angew. Chem., Int. Ed..

[59] Zheng C., Mao Y., Kozuch J., Atsango A. O., Ji Z., Markland T. E., Boxer S. G. (2022). A Two-Directional
Vibrational Probe Reveals Different
Electric Field Orientations in Solution and an Enzyme Active Site. Nat. Chem..

[60] Fried S. D., Boxer S. G. (2017). Electric Fields and Enzyme Catalysis. Annu. Rev. Biochem..

[61] Siddiqui S. A., Stuyver T., Shaik S., Dubey K. D. (2023). Designed Local Electric
Fields–Promising Tools for Enzyme Engineering. JACS Au.

[62] Huang, X. ; Tang, C. ; Li, J. ; Chen, L. C. ; Zheng, J. ; Zhang, P. ; Le, J. ; Li, R. ; Li, X. ; Liu, J. ; Yang, Y. ; Shi, J. ; Chen, Z. ; Bai, M. ; Zhang, H. L. ; Xia, H. ; Cheng, J. ; Tian, Z. Q. ; Hong, W. Electric Field-Induced Selective Catalysis of Single-Molecule Reaction. Sci. Adv. 2019, 5 (6). 10.1126/sciadv.aaw3072. PMC6588380 31245539

[63] Aragones A. C., Haworth N. L., Darwish N., Ciampi S., Mannix E. J., Wallace G. G., Diez-Perez I., Coote M. L. (2016). Electrostatic Catalysis
of a Diels–Alder Reaction. Nature.

[64] Makhnev V. Y., Kyuberis A. A., Zobov N. F., Lodi L., Tennyson J., Polyansky O. L. (2018). High Accuracy
Ab Initio Calculations of Rotational-Vibrational
Levels of the HCN/HNC System. J. Phys. Chem.
A.

[65] Hirota T., Yamamoto S., Kawaguchi K., Sakamoto A., Ukita N. (1999). Observations
of HCN, HNC, and NH3 in Comet Hale-Bopp. Astrophys.
J..

[66] Schmidt D. R., Ziurys L. M. (2017). New Detections of HNC in Planetary Nebulae: Evolution
of the [HCN]/[HNC] Ratio. Astrophys. J..

[67] Jin M., Lee J. E., Kim K. T. (2015). The HCN/HNC
Abundance Ratio Toward
Different Evolutionary Phases of Massive Star Formation. Astrophys J. Suppl Ser..

[68] Padovani M., Walmsley C. M., Tafalla M., Hily-Blant P., Pineau des Forets G. (2011). Hydrogen Cyanide and Isocyanide in Prestellar Cores. Astronomy & Astrophysics.

[69] Graninger D., Öberg K. I., Qi C., Kastner J. (2015). HNC In Protoplanetary
Disks. Astrophysical Journal Letters.

[70] Irvine W. M., Bockelee-Morvan D., Lis D. C., Matthews H. E., Biver N., Crovisier J., Davies J. K., Dent W. R. F., Gautier D., Godfrey P. D., Keene J., Lovell A. J., Owen T. C., Phillips T. G., Rauer H., Schloerb F. P., Senay M., Young K. (1996). Spectroscopic
Evidence for Interstellar Ices in Comet Hyakutake. Nature.

[71] Irvine W. M., Dickens J. E., Lovell A. J., Schloerb F. P., Senay M., Bergin E. A., Jewitt D., Matthews H. E. (1997). The HNC/HCN Ratio
in Comets. Earth Moon Planets.

[72] Biver N., Bockelée-Morvan D., Crovisier J., Lis D. C., Moreno R., Colom P., Henry F., Herpin F., Paubert G., Womack M. (2006). Radio Wavelength Molecular
Observations of Comets C/1999 T1 (McNaught-Hartley), C/2001 A2 (LINEAR),
C/2000 WM1 (LINEAR) and 153P/Ikeya-Zhang. Astron.
Astrophys..

[73] Agúndez M., Biver N., Santos-Sanz P., Bockelée-Morvan D., Moreno R. (2014). Molecular Observations
of Comets C/2012 S1 (ISON) and
C/2013 R1 (Lovejoy): HNC/HCN Ratios and Upper Limits to PH3. Astron. Astrophys..

[74] Irvine W. M., Bergman P., Lowe T. B., Matthews H., McGonagle D., Nummelin A., Owen T. (2003). HCN and HNC in Comets C/2000 WM1
(Linear) and C/2002 C1 (Ikeya-Zhang). Origins
of Life and Evolution of the Biosphere.

[75] Biver N., Bockelée-Morvan D., Colom P., Crovisier J., Davies J. K., Dent W. R. F., Despois D., Gérard E., Lellouch E., Rauer H., Moreno R., Paubert G. (1997). Evolution
of the Outgassing of Comet Hale-Bopp (C/1995 O1) from Radio Observations. Science.

[76] Rodgers S. D., Butner H. M., Charnley S. B., Ehrenfreund P. (2003). The HNC/HCN
Ratio in Comets: Observations of C/2002 C1 (Ikeya-Zhang). Adv. Space Res..

[77] Harada N., Saito T., Nishimura Y., Watanabe Y., Sakamoto K. (2024). A Temperature
or Far-Ultraviolet Tracer? The HNC/HCN Ratio in M83 on the Scale of
Giant Molecular Clouds. Astrophys. J..

[78] Moreno R., Lellouch E., Lara L. M., Courtin R., Bockelée-Morvan D., Hartogh P., Rengel M., Biver N., Banaszkiewicz M., González A. (2011). First Detection of Hydrogen Isocyanide (HNC) in Titan’s
Atmosphere. Astron. Astrophys..

[79] Rodgers S. D., Charnley S. B. (2001). On the Origin of
HNC in Comet Lee. Mon. Not. R. Astron. Soc..

[80] Loison J. C., Wakelam V., Hickson K. M. (2014). The Interstellar
Gas-Phase Chemistry
of HCN and HNC. Mon. Not. R. Astron. Soc..

[81] Chen H. F., Liu M. C., Chen S. C., Huang T. P., Wu Y. J. (2015). Irradiation
of Ethylene Diluted in Solid Nitrogen with Vacuum Ultraviolet Light
and Electrons: Its Implications for the Formation of HCN and HNC. Astrophys. J..

[82] Enrique-Romero J., Lamberts T. (2024). The Complex (Organic) Puzzle of the
Formation of Hydrogen
Cyanide and Isocyanide on Interstellar Ice Analogues. J. Phys. Chem. Lett..

[83] Baiano C., Lupi J., Barone V., Tasinato N. (2022). Gliding on Ice in Search
of Accurate and Cost-Effective Computational Methods for Astrochemistry
on Grains: The Puzzling Case of the HCN Isomerization. J. Chem. Theory Comput.

[84] Koch D. M., Toubin C., Xu S., Peslherbe G. H., Hynes J. T. (2007). Concerted Proton-Transfer Mechanism
and Solvation Effects
in the HNC/HCN Isomerization on the Surface of Icy Grain Mantles in
the Interstellar Medium. J. Phys. Chem. C.

[85] Khalouf-Rivera J., Carvajal M., Santos L. F., Pérez-Bernal F. (2019). Calculation
of Transition State Energies in the HCN-HNC Isomerization with an
Algebraic Model. J. Phys. Chem. A.

[86] Zamir A., Stein T. (2022). Isomerization of Hydrogen Cyanide and Hydrogen Isocyanide in a Cluster
Environment: Quantum Chemical Study. J. Chem.
Phys..

[87] DePrince A. E., Mazziotti D. A. (2008). Molecular Geometries and Harmonic Frequencies from
the Parametric Two-Electron Reduced Density Matrix Method with Application
to the HCN ↔ HNC Isomerization. J. Phys.
Chem. B.

[88] Cotton C. E., Francisco J. S., Klemperer W. (2013). Computational Study of the Linear
Proton Bound Ion–Molecule Complexes of HCNH+ with HCN and
HNC. J. Chem. Phys..

[89] Brown W. L., Lanzerotti L. J., Johnson R. E. (1982). Fast Ion Bombardment of Ices and
Its Astrophysical Implications. Science.

[90] Petrie S. (2001). Hydrogen Isocyanide,
HNC: A Key Species in the Chemistry of Titan’s Ionosphere?. Icarus.

[91] Loison J. C., Hébrard E., Dobrijevic M., Hickson K. M., Caralp F., Hue V., Gronoff G., Venot O., Bénilan Y. (2015). The Neutral
Photochemistry of Nitriles, Amines and Imines in the Atmosphere of
Titan. Icarus.

[92] Molina-Cuberos G. J., López-Moreno J. J., Rodrigo R., Lara L. M., O’Brien K. (1999). Ionization
by Cosmic Rays of the Atmosphere of Titan. Planet
Space Sci..

[93] Cordiner M. A., Nixon C. A., Teanby N. A., Irwin P. G. J., Serigano J., Charnley S. B., Milam S. N., Mumma M. J., Lis D. C., Villanueva G., Paganini L., Kuan Y. J., Remijan A. J. (2014). ALMA Measurements
of the HNC and HC3N Distributions in Titan’s Atmosphere. Astrophys J. Lett..

[94] Wulff G. (1901). XXV. Zur Frage
Der Geschwindigkeit Des Wachsthums Und Der Auflösung Der Krystallflächen. Z. Kristallogr Cryst. Mater..

[95] Laue M. (1943). v. Der Wulffsche
Satz Für Die Gleichgewichtsform von Kristallen. Z. Kristallogr Cryst. Mater..

[96] Li R., Zhang X., Dong H., Li Q., Shuai Z., Hu W. (2016). Gibbs–Curie–Wulff Theorem
in Organic Materials: A Case
Study on the Relationship between Surface Energy and Crystal Growth. Adv. Mater..

[97] Hesterberg R., Macchi P., Hulliger J. (2018). Polarization,
Inner and Outer Field,
and Surface Charge Compensation of a Molecular Crystal. Cryst. Growth Des.

[98] Du M. H., Zhang S. B., Northrup J. E., Erwin S. C. (2008). Stabilization Mechanisms
of Polar Surfaces: ZnO Surfaces. Phys. Rev.
B Condens Matter Mater. Phys..

[99] Goniakowski J., Finocchi F., Noguera C. (2008). Polarity of
Oxide Surfaces and Nanostructures. Rep. Prog.
Phys..

[100] Dachev T. P. (2013). Profile of the Ionizing Radiation
Exposure between
the Earth Surface and Free Space. J. Atmos Sol
Terr Phys..

[101] Li H., Su T. A., Zhang V., Steigerwald M. L., Nuckolls C., Venkataraman L. (2015). Electric Field
Breakdown in Single
Molecule Junctions. J. Am. Chem. Soc..

[102] Zang Y., Zou Q., Fu T., Ng F., Fowler B., Yang J., Li H., Steigerwald M. L., Nuckolls C., Venkataraman L. (2019). Directing Isomerization Reactions
of Cumulenes with Electric Fields. Nat. Commun..

[103] Ji Z., Kozuch J., Mathews I. I., Diercks C. S., Shamsudin Y., Schulz M. A., Boxer S. G. (2022). Protein Electric Fields Enable Faster
and Longer-Lasting Covalent Inhibition of β-Lactamases. J. Am. Chem. Soc..

[104] Hunter E. P. L., Lias S. G. (1998). Evaluated Gas Phase
Basicities and
Proton Affinities of Molecules: An Update. J.
Phys. Chem. Ref. Data.

[105] Lias, S. G. ; Bartmess, J. E. ; Liebman, J. F. ; Holmes, J. L. ; Levin, R. D. ; Mallard, W. G. Gas-Phase Ion and Neutral Thermochemistry. J. Phys. Chem. Ref. Data; American Chemical Society and the American Institute of Physics, 1988; Vol. 17.

[106] Coates, A. J. ; Crary, F. J. ; Lewis, G. R. ; Young, D. T. ; Waite, J. H. ; Sittler, J. C. Discovery of Heavy Negative Ions in Titan’s Ionosphere. Geophys. Res. Lett. 2007, 34 (22). 10.1029/2007GL030978.

[107] Vuitton V., Lavvas P., Yelle R. V., Galand M., Wellbrock A., Lewis G. R., Coates A. J., Wahlund J. E. (2009). Negative
Ion Chemistry in Titan’s Upper Atmosphere. Planet Space Sci..

[108] Cruz-Diaz G. A, Martin-Domenech R., Moreno E., Munoz Caro G. M, Chen Y.-J. (2018). New Measurements
on Water Ice Photodesorption and Product
Formation under Ultraviolet Irradiation. Mon.
Not. R. Astron. Soc..

[109] Dupuy R., Bertin M., Féraud G., Michaut X., Jeseck P., Doronin M., Philippe L., Romanzin C., Fillion J. H. (2017). Spectrally-Resolved UV Photodesorption
of CH4 in Pure and Layered Ices. Astron. Astrophys..

[110] Öberg K. I., Van Dishoeck E. F., Linnartz H. (2009). Photodesorption of
Ices I: CO. And. Astron Astrophys.

[111] Paardekooper D. M., Fedoseev G., Riedo A., Linnartz H. (2016). A Novel Approach
to Measure Photodesorption Rates of Interstellar Ice Analogues - The
Photodesorption Rate of CO Ice Reinvestigated. Astron. Astrophys..

[112] Irvine W. M., Bergin E. A., Dickens J. E., Jewitt D., Lovell A. J., Matthews H. E., Schloerb F. P., Senay M. (1998). Chemical Processing
in the Coma as the Source of Cometary HNC. Nature.

[113] Johnson R.
E., Brown W. L., Lanzerotti L. J. (1983). Energetic
Charged Particle Erosion of Ices in the Solar System. J. Phys. Chem..

[114] Kresse G., Furthmüller J. (1996). Efficient
Iterative Schemes for Ab
Initio Total-Energy Calculations Using a Plane-Wave Basis Set. Phys. Rev. B.

[115] Blöchl P. E. (1994). Projector Augmented-Wave Method. Phys. Rev. B.

[116] Grimme S., Ehrlich S., Goerigk L. (2011). Effect of the Damping
Function in Dispersion Corrected Density Functional Theory. J. Comput. Chem..

[117] Makov G., Payne M. C. (1995). Periodic Boundary
Conditions in Ab
Initio Calculations. Phys. Rev. B.

[118] Virtanen P., Gommers R., Oliphant T. E., Haberland M., Reddy T., Cournapeau D., Burovski E., Peterson P., Weckesser W., Bright J., van der Walt S. J., Brett M., Wilson J., Millman K. J., Mayorov N., Nelson A. R. J., Jones E., Kern R., Larson E., Carey C J, Polat I., Feng Y., Moore E. W., VanderPlas J., Laxalde D., Perktold J., Cimrman R., Henriksen I., Quintero E. A., Harris C. R., Archibald A. M., Ribeiro A. H., Pedregosa F., van Mulbregt P., Vijaykumar A., Bardelli A. P., Rothberg A., Hilboll A., Kloeckner A., Scopatz A., Lee A., Rokem A., Woods C. N., Fulton C., Masson C., Haggstrom C., Fitzgerald C., Nicholson D. A., Hagen D. R., Pasechnik D. V., Olivetti E., Martin E., Wieser E., Silva F., Lenders F., Wilhelm F., Young G., Price G. A., Ingold G.-L., Allen G. E., Lee G. R., Audren H., Probst I., Dietrich J. P., Silterra J., Webber J. T, Slavic J., Nothman J., Buchner J., Kulick J., Schonberger J. L., de Miranda Cardoso J.
V., Reimer J., Harrington J., Rodriguez J. L. C., Nunez-Iglesias J., Kuczynski J., Tritz K., Thoma M., Newville M., Kummerer M., Bolingbroke M., Tartre M., Pak M., Smith N. J., Nowaczyk N., Shebanov N., Pavlyk O., Brodtkorb P. A., Lee P., McGibbon R. T., Feldbauer R., Lewis S., Tygier S., Sievert S., Vigna S., Peterson S., More S., Pudlik T., Oshima T., Pingel T. J., Robitaille T. P., Spura T., Jones T. R., Cera T., Leslie T., Zito T., Krauss T., Upadhyay U., Halchenko Y. O., Vazquez-Baeza Y. (2020). SciPy 1.0:
Fundamental Algorithms for Scientific Computing in Python. Nat. Methods.

[119] Rahm J. M., Erhart P. (2020). WulffPack: A Python
Package for Wulff
Constructions. Journal of Open Source Software.

[120] Frisch, M. J. ; et al. Gaussian 16, Revision B.01; Gaussian: Wallingford, CT, 2016.

[121] Neese, F. Software Update: The ORCA Program System—Version 6.0. Wiley Interdiscip. Rev. Comput. Mol. Sci. 2025, 15 (2). 10.1002/wcms.70019.

[122] Riplinger, C. ; Neese, F. An Efficient and near Linear Scaling Pair Natural Orbital Based Local Coupled Cluster Method. J. Chem. Phys. 2013, 138 (3). 10.1063/1.4773581. 23343267

[123] Grimme S. (2012). Supramolecular Binding Thermodynamics
by Dispersion-Corrected
Density Functional Theory. Chemistry –
A European Journal.

