# Supplementary material for: Electric Fields Can Assist Prebiotic Reactivity on Hydrogen Cyanide Surfaces
Source: ACS Cent Sci. 2026 Jan 14;12(1):111–21. doi: 10.1021/acscentsci.5c01497 (PMC12856676; doi:10.1021/acscentsci.5c01497)
Supplement: Supplementary file 1 [file oc5c01497_si_001.pdf]

Supporting Information for

Electric Fields Can Assist Prebiotic Reactivity on Hydrogen Cyanide Surfaces

Marco Cappelletti[1], Hilda Sandström[1,†], and Martin Rahm[1,*]

[1]Department of Chemistry and Chemical Engineering, Chalmers University of Technology, Gothenburg 412 96, Sweden

†Present address: Department of Chemistry, Technical University of Munich, James-Franck-Str. 1, 85748, Garching b. Munich, Germany

*Corresponding author. E-mail: martin.rahm@chalmers.se

Table of contents

1. HCN crystal properties **3**

1.1 Lattice parameters 3

1.2 Surface energies 3

2. Wulff constructions **4**

2.1 Aspect ratio 4

2.2 Surface energy interpolation 5

2.3 Best estimate of the single crystal 5

3. Cooperative effects of linear HCN clusters **7**

3.1 Electric fields 7

3.2 Chemical properties of HCN clusters 7

3.3 Energetics of the isomerization mechanisms 8

3.4 Dissociation of HCN and HNC into gas-phase from HCN clusters 9

4. References **10**

Dataset

The dataset containing optimized structures, VASP 6.4.1, Gaussian 16, and ORCA 6 input and output files are available at the Swedish National Data (SND) Service at the following link:

https://doi.org/10.71870/m5z3-m463

1. HCN crystal properties

1.1 Lattice parameters

Calculated lattice parameters for the *Imm2* orthorhombic structure of Hydrogen Cyanide (HCN) (Table 1) are in close agreement with experiments. A ~2% discrepancy is attributed to thermal lattice expansion omitted from our 0 K calculations.

Table S1: Lattice parameters for the orthorhombic phase of the HCN crystal. [a] From Ref. [1].

	a	b	c
This work (0 K)	4.02	4.76	4.25
Exp. (153 K)[a]	4.13±0.02	4.85±0.02	4.34±0.02

1.2 Surface energies

Our computed surface energies (γ) are reported in Table S2. These values were obtained from surface energies calculated for slabs of increasing thicknesses n (up to 20 molecular layers), $\gamma_{hkl}(n)$, and extrapolated to the limit $n\rightarrow\infty$. The computed data were fitted according to the function:

$$\gamma_{hkl}(n) = \frac{n \cdot \gamma_{hkl} + a}{n + b} \quad \text{(S1)}$$

Where γ_{hkl}, a, and b are the fitting parameters, determined using the least square method (Figure S1). γ_{hkl} is the surface energy at infinite slab thickness.

Table S2: Predicted surface energies (γ) of the HCN crystal. Lattice planes are represented by a set of Miller indices. For polar surfaces, γ represent the average energy of the two opposing surfaces, {hkl} and $\{hk\bar{l}\}$, of the crystal.

**Surfaces**	**Energy, γ_{hkl} (J/m^2)**
**Nonpolar**	
{100}	0.075
{010}	0.079
{110}	0.066
{120}	0.075
{210}	0.074
**Polar**	
$\{001\}/\{00\bar{1}\}$	1.65
$\{011\}/\{01\bar{1}\}$	1.25
$\{101\}/\{10\bar{1}\}$	1.16
$\{111\}/\{11\bar{1}\}$	1.02
$\{201\}/\{20\bar{1}\}$	0.86
$\{021\}/\{02\bar{1}\}$	0.96
$\{102\}/\{10\bar{2}\}$	1.47
$\{012\}/\{01\bar{2}\}$	1.46
$\{013\}/\{01\bar{3}\}$	1.60
$\{013\}/\{10\bar{3}\}$	1.58
$\{112\}/\{11\bar{2}\}$	1.39

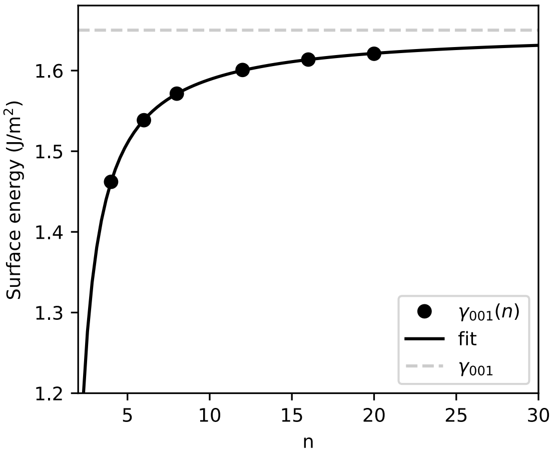

Figure S1: Example of surface energy determination for the $\{001\}/\{00\bar{1}\}$ surfaces. Computed data ($\gamma_{001}(n)$), are plotted against the slab thickness *n*, and fitted using the function in equation S1 (black line). The dashed grey line is the extrapolated value for $n \rightarrow \infty$, γ_{001} = 1.65 J/m^2 (Table S2).

2. Wulff constructions

2.1 Aspect ratio

Our predicted HCN single crystals feature a rounded rhombus section and truncated square pyramid terminations (Figure S2). Such shape makes it difficult to uniquely define an aspect ratio (AR). The Wulffpack python package [2], which employes the Atomic Simulation Environment (ASE) package [3], provides the volume *V* of the single crystal, allowing us to estimate the area *A* of the rounded rhombus as:

$$A \approx \frac{V}{\Delta z} \qquad \text{(S2)}$$

Where *Δz* is the height (the longest dimension) of the Wulff construction. The aspect ratio *AR* can then be estimated using the equation:

$$AR = \frac{\Delta z}{\sqrt{A}} \approx \sqrt{\frac{\Delta z^3}{V}} \qquad \text{(S3)}$$

The aspect ratio does not vary substantially with respect to the size of the crystal, and converges to a value of circa 25.95 (Figure S3).

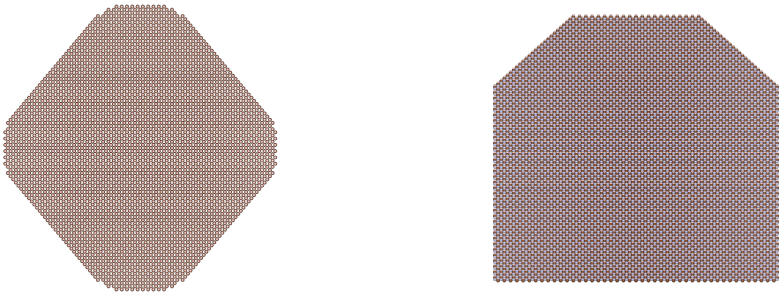

Figure S2: Top-view (left) and side-view (right) of the HCN Wulff construction tip.

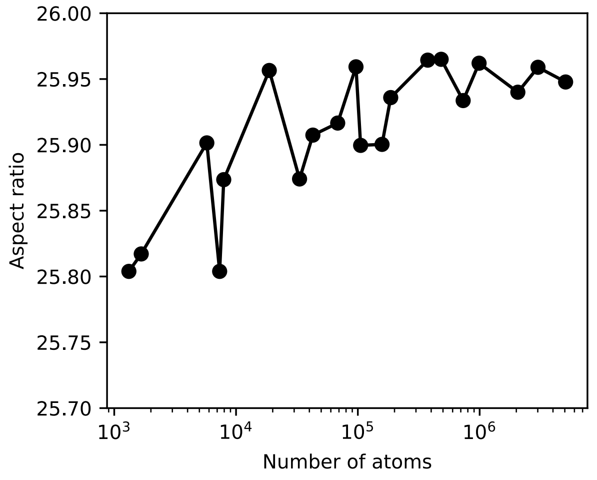

Figure S3: Aspect ratio of Wulff constructions with respect to the number of atoms in the crystal.

2.2 Surface energy interpolation

To refine our prediction of the crystal morphology, we estimated the energies of additional surfaces by interpolating our computed surface energies (γ) in the Miller vector space. Specifically, we considered all surfaces with Miller indices satisfying $|h| + |k| + |l| \leq 20$, resulting in a total of 2633 orientations. This interpolation approach is justified by the observation that surface energy (γ) varies smoothly across Miller space (Figure S4), making it suitable for estimating values beyond those we computed.

In our method, each (h,k,l) surface was represented by a vector v_{hkl} = (h,k,l), which was normalized to obtain v_{xyx} = (x,y,z) = (h,k,l)/$||v_{hkl}||$. For instance, the (0,1,1) lattice plane corresponds to the vector $v_{011} = (0,\sqrt{2}, \sqrt{2})$. This normalization projects each orientation onto a point on the surface of a unit sphere, restricted to one octant. These points were then associated with their corresponding computed surface energies γ_{hkl}. Interpolation over this spherical surface allows estimation of γ for arbitrary directions within the octant, effectively enabling prediction of γ_{hkl} for any set of Miller indices. The numerical interpolation was carried out using the radial basis function (Rbf) method, as implemented in the python package scipy, version 1.15.2 [4].

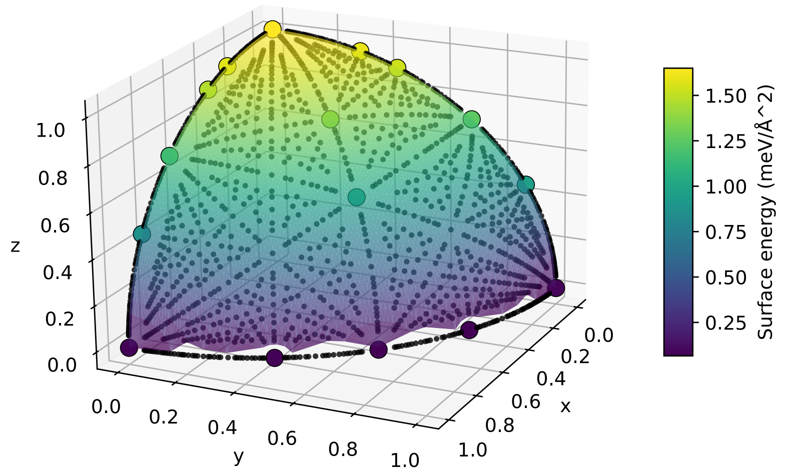

Figure S4: Computed surface energies γ_{hkl} (colored circles) and their interpolation (colored surface) in the space defined by normalized Miller vectors. Black dots represent the additional data ($|h|+|k|+|l| \leq 20$) used to build our best estimate of the HCN single crystal.

2.3 Best estimate of the single crystal

Using the interpolated surface energy values, we obtained our best estimate of the HCN crystal morphology. The fractional coverages of the exposed surfaces in a Wulff construction composed of ~$1 \cdot 10^7$ atoms (Table S3) reveal that non-polar surfaces make up 92.9% of the total surface area. The most represented polar surfaces are the highly slanted $\{9,9,1\}/\{9,9,\bar{1}\}$ and $\{8,9,2\}/\{8,9,\bar{2}\}$ facets, which give rise to chemically active step edges near the crystal tip (Figure S5).

Table S3: Fractional coverages, expressed in percentage of the total surface area of the crystal, of the {h,k,l} surfaces in our best estimate of the HCN single crystal, composed of ~$1\cdot10^7$ atoms. Shown are the 67 facets (out of 2633) with non-zero surface coverage.

Surface	Coverage	Surface	Coverage	Surface	Coverage
{1,1,0}	21.7%	{3,0,$\bar{4}$}	0.22%	{8,9,1}	0.07%
{13,2,0}	12.1%	{3,0,4}	0.22%	{4,5,1}	0.05%
{2,13,0}	10.2%	{7,7,1}	0.21%	{4,5,$\bar{1}$}	0.05%
{6,1,0}	10.0%	{7,7,$\bar{1}$}	0.21%	{3,16,$\bar{1}$}	0.05%
{1,6,0}	8.6%	{7,0,9}	0.19%	{3,16,1}	0.05%
{1,7,0}	6.4%	{7,0,$\bar{9}$}	0.19%	{0,7,9}	0.04%
{7,1,0}	5.5%	{9,9,2}	0.16%	{0,7,$\bar{9}$}	0.04%
{3,17,0}	5.2%	{9,9,$\bar{2}$}	0.16%	{8,0,11}	0.03%
{17,3,0}	3.7%	{8,8,1}	0.15%	{8,0,$\overline{11}$}	0.03%
{2,11,0}	3.5%	{8,8,$\bar{1}$}	0.15%	{4,0,$\bar{5}$}	0.02%
{3,16,0}	2.3%	{1,8,$\overline{10}$}	0.14%	{4,0,5}	0.02%
{10,9,0}	2.0%	{1,8,10}	0.14%	{4,4,1}	0.02%
{9,10,0}	1.9%	{5,6,1}	0.14%	{4,4,$\bar{1}$}	0.02%
{9,9,1}	0.48%	{5,6,$\bar{1}$}	0.14%	{0,5,$\bar{6}$}	0.02%
{9,9,$\bar{1}$}	0.48%	{6,7,1}	0.13%	{0,5,6}	0.02%
{8,9,2}	0.31%	{6,7,$\bar{1}$}	0.13%	{0,0,1}	0.02%
{8,9,$\bar{2}$}	0.31%	{7,8,$\bar{1}$}	0.09%	{0,0,$\bar{1}$}	0.02%
{6,6,1}	0.31%	{7,8,1}	0.09%	{7,8,$\bar{2}$}	0.004%
{6,6,$\bar{1}$}	0.31%	{0,4,5}	0.08%	{7,8,2}	0.004%
{9,10,1}	0.28%	{0,4,$\bar{5}$}	0.08%	{9,8,$\bar{2}$}	0.001%
{9,10,$\bar{1}$}	0.28%	{0,9,11}	0.07%	{9,8,2}	0.001%
{5,5,$\bar{1}$}	0.28%	{0,9,$\overline{11}$ }	0.07%		
{5,5,1}	0.28%	{8,9,$\bar{1}$}	0.07%		

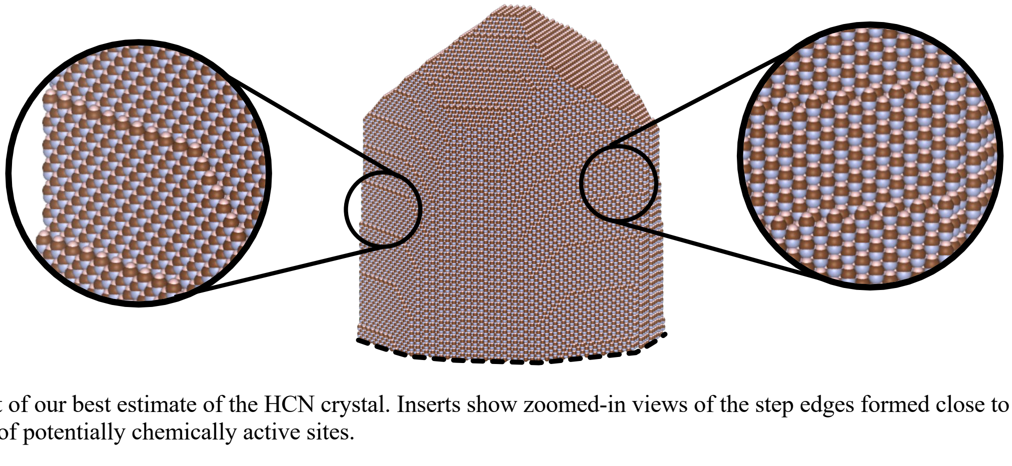

Figure S5: Cut of our best estimate of the HCN crystal. Inserts show zoomed-in views of the step edges formed close to the tip, which are indicative of potentially chemically active sites.

3. Cooperative effects of linear HCN clusters

3.1 Electric fields

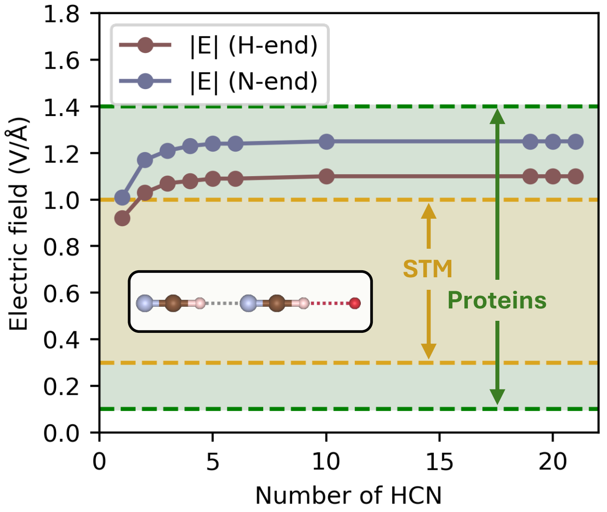

Figure S6. Computed magnitude of the electric field generated at the 1.9 Å hydrogen bonding distance from the H-end (brown circles) and from the N-end (pale blue circles) of a linear HCN chain. Insert shows as an example the point of calculation from the H-end as a red sphere. Predicted field strengths are sufficiently large to affect chemical change. The range of electric field magnitudes [5] present inside proteins and in a scanning tunneling microscopy (STM) are shown in green and yellow, respectably, for comparison.

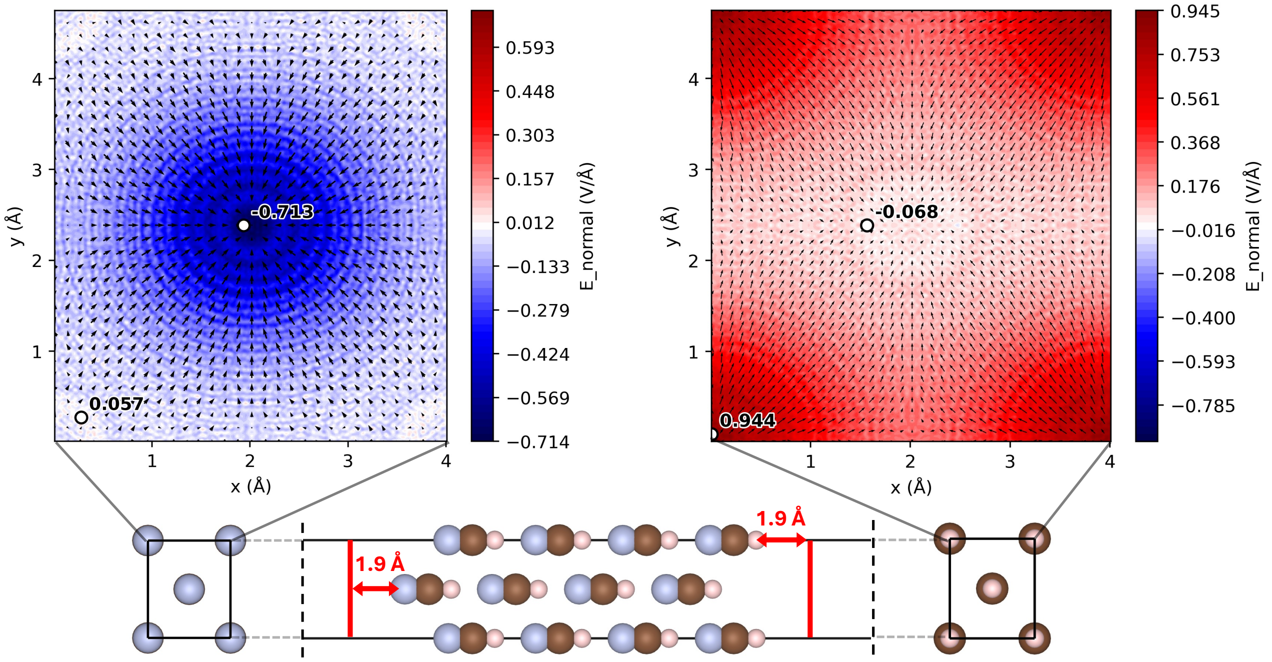

Figure S7. 2D plots of the electric field intensity at 1.9 Å from the N-end (left) and from the H-end (right) surface of a 4-HCN-unit (001) slab. The contour represents the out-of-plane (normal) component of the electric field, and overlayed is a quiver plot of the in-plane projected electric field vectors. Positive values (in red) indicate a normal component exiting the plane, while negative values (in blue) indicate a normal component entering the plane. Labeled data points show the minimum and the maximum value of the normal component. The field appears somewhat dampened compared to the linear chain, especially at the N-end.

3.2 Chemical properties of HCN clusters

The proton affinity (PA) and the gas-phase basicity (GB) of a species M are defined as the negative of the enthalpy change, -ΔH, and the negative of the Gibbs energy change, -ΔG, respectively, of the reaction:

$$M + H^+ \rightarrow MH^+ \qquad \text{(S4)}$$

The gas-phase acidity (GA) of a species XH is defined as the Gibbs energy change, ΔG, of the reaction:

$$XH \rightarrow X^- + H^+ \qquad (S5)$$

While protonation, as described by PA and GB, is typically a favorable process, the deprotonation of a neutral species is generally endergonic, resulting in a positive GA. Thus, lower GA values indicate that the deprotonation is less disfavored, i.e. the species is more acidic. For example, the GA of ethanol is 1551±8 kJ/mol, while that of HBr is 1331±1 kJ/mol [6].

Our predictions (see the Methods section) at 90 K, the surface temperature of Titan, and 259 K, near the melting point of HCN, for HCN in vacuum and for a linear HCN chain composed of 20 units are presented in Table S4. As method validation, we also report values at 298.15 K for gas-phase HCN, which show remarkable agreement with experimental estimates.

Table S4: Gas-phase proton affinity (PA), gas-phase basicity (GB), and gas-phase acidity (GA) of HCN (n=1) and a $(HCN)_{20}$ linear chain (n=20) at 90 K (surface of Titan) and 259 K (near the melting point of HCN). Values in square brackets denote change in percentage relative to the single molecule. Data for gas-phase HCN at room temperature (298.15 K) is compared to experimental estimates. [a] Note that the protonated chain spontaneously and without barrier shuttles a proton such that an HNC is generated at the surface and an $HCNH^+$ inside the chain (see Figure 5 of main text). [b] From Ref. [6]. [c] From Ref. [7].

$(HCN)_n$	PA[a] (kJ/mol)	GB[a] (kJ/mol)	GA (kJ/mol)
**90 K**			
n = 1	708	700	1456
n = 20	845 [+19%]	834 [+19%]	1322 [-9%]
**259 K**			
n = 1	711	684	1440
n = 20	850 [+20%]	812 [+19%]	1310 [-9%]
**298.15 K**			
n = 1	711	680	1436
Exp.	712.9[b]	681.6[b]	1438±8[c]

3.3 Energetics of the isomerization mechanisms

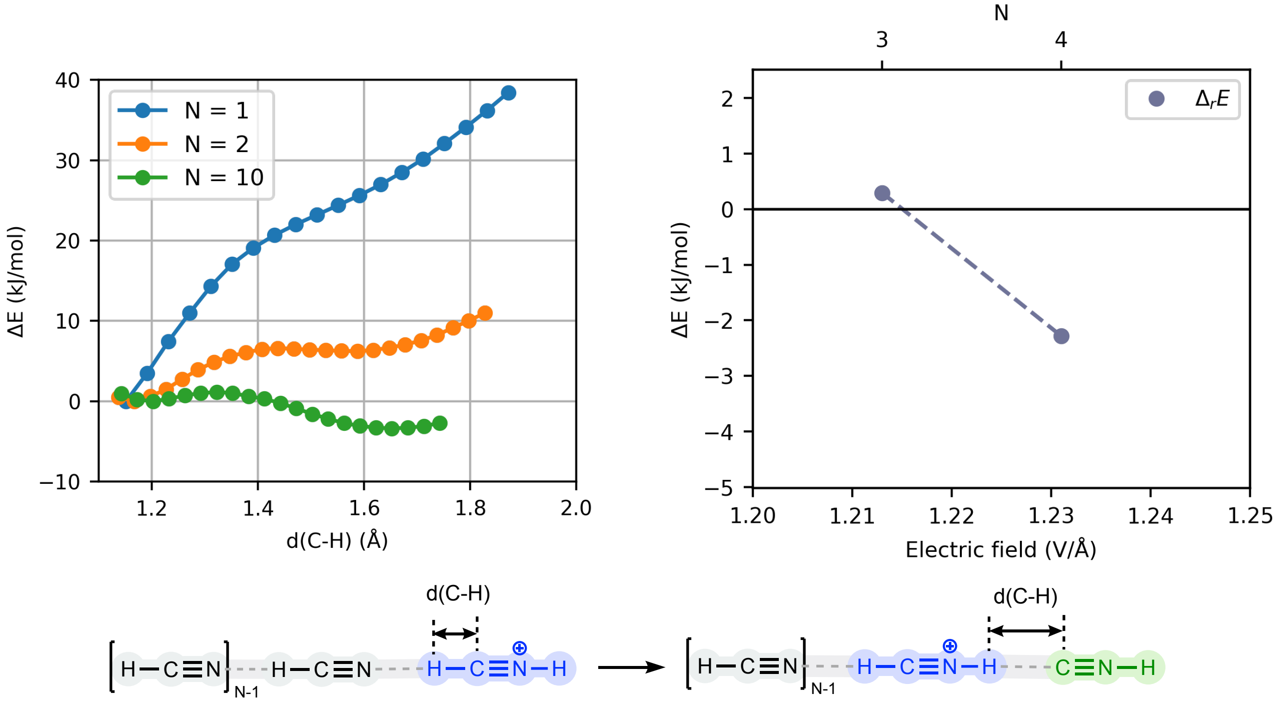

Figure S8. Energetics of the cationic HCN to HNC isomerization mechanism. Left plot: Relaxed scan of the proton transfer, isolated (N=1, in blue), or catalyzed by either a single HCN (N=2, orange), or a 9-HCN-unit chain (N=10, green). Plotted are DLPNO-CCSD(T)/aug-cc-pVTZ//B3LYP-D3(BJ)/ 6-311++G(d,p) electronic energies against C-H distance, as shown in the bottom scheme. Right plot: Reaction energy ($\Delta_r E$) (DLPNO-CCSD(T)/aug-cc-pVTZ//B3LYP-D3(BJ)/ 6-311++G(d,p) electronic energies) of the mechanism against the electric field at 1.9 Å from the N-end of the linear chain (see Figure S6). The length of the chain (N) is also shown in the upper horizontal axis. For N<3, the product state is not a minimum in the DFT PES; conversely, for N>4, the reactant state is not a minimum in the DFT PES. Transition states could not be identified for any of these reactions.

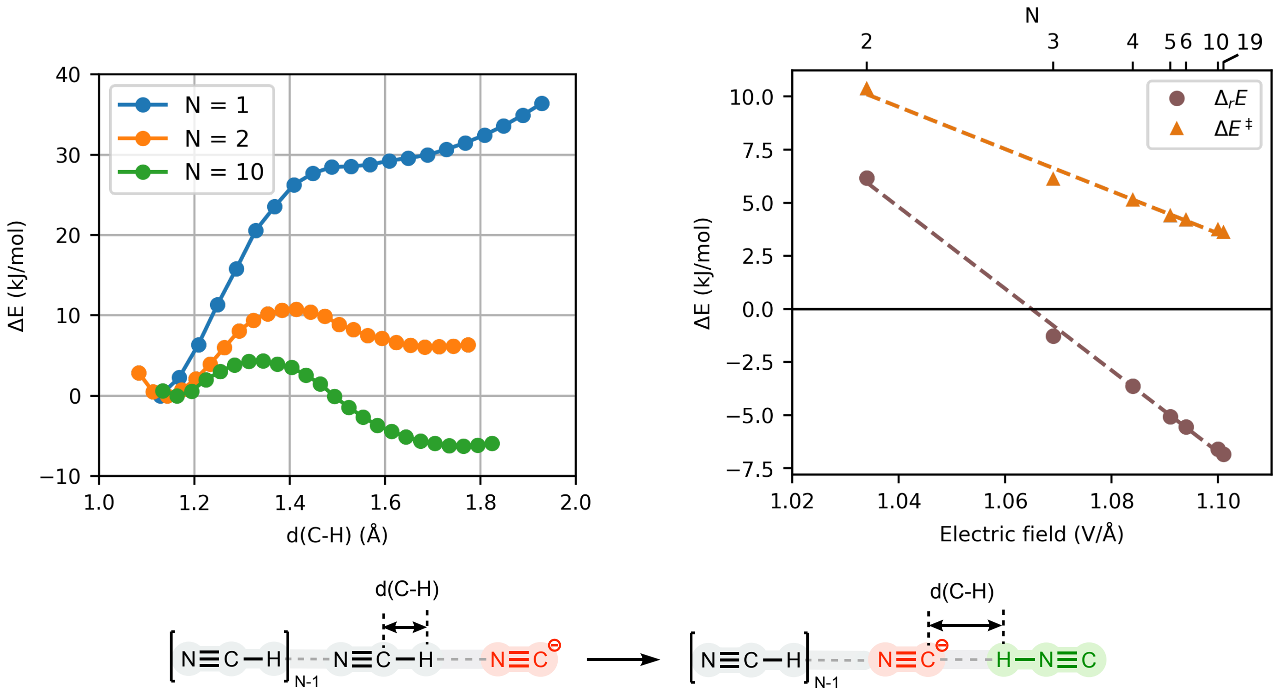

Figure S9. Energetics of the anionic HCN to HNC isomerization mechanism. Left plot: Relaxed scan of the proton transfer, isolated (N=1, in blue), or catalyzed by either a single HNC (N=2, orange) or a 9-HCN-unit chain (N=10, green). Plotted are DLPNO-CCSD(T)/aug-cc-pVTZ//B3LYP-D3(BJ)/ 6-311++G(d,p) electronic energies against C-H distance, as shown in the bottom scheme. Right plot: Reaction energy (Δ_rE) and reaction barrier ($\Delta E^{\ddagger}$) (DLPNO-CCSD(T)/aug-cc-pVTZ//B3LYP-D3(BJ)/ 6-311++G(d,p) electronic energies) of the mechanism against the electric field at 1.9 Å from the H-end of the linear chain (see Figure S6). The length of the chain (N) is also shown in the upper horizontal axis. The uncatalyzed isomerization mechanism is unfavored and the product is not a minimum in the potential energy surface. The isomerization becomes favored as N≥3. Both the reaction energy and the barrier decrease linearly with the intensity of the electric field.

3.4 Dissociation of HCN and HNC into gas-phase from HCN clusters

In our proposed HCN isomerization mechanism catalyzed by surface ionization, the removal of a terminal HNC molecule represents the rate determining step. To evaluate the feasibility of this process, we computed the DLPNO-CCSD(T)-refined gas-phase dissociation energies (ΔG) of HNC and, for comparison, HCN from a linear HCN chain in its neutral, protonated, or deprotonated form (Figures S10). Gibbs energies were computed at 90 K, and 180 K, the temperature at the surface and in the atmosphere of Titan, respectively, and at 259 K, near the melting point of HCN.

Our calculations indicate that HNC forms stronger intermolecular interactions through its hydrogen end compared to HCN. HNC binds more strongly to the N-terminated end of the neutral chain (26 kJ/mol vs 15 kJ/mol at 90 K) and to the negatively charged chain (75 kJ/mol vs 47 kJ/mol at 90 K). In contrast, the energies of dissociation for HNC and HCN are similar when these molecules are bound to positively charged terminations.

For charged chains, the gas-phase dissociation energy of HNC is 75 kJ/mol at 90 K and 52-53 kJ/mol at 259 K. While these barriers are too high to be overcome by thermal motion alone, non-thermal processes, such as ionization events or chemical interactions with other species, may enable the self-propagation of the isomerization cycle, ultimately contributing to the depletion of HCN from the surface.

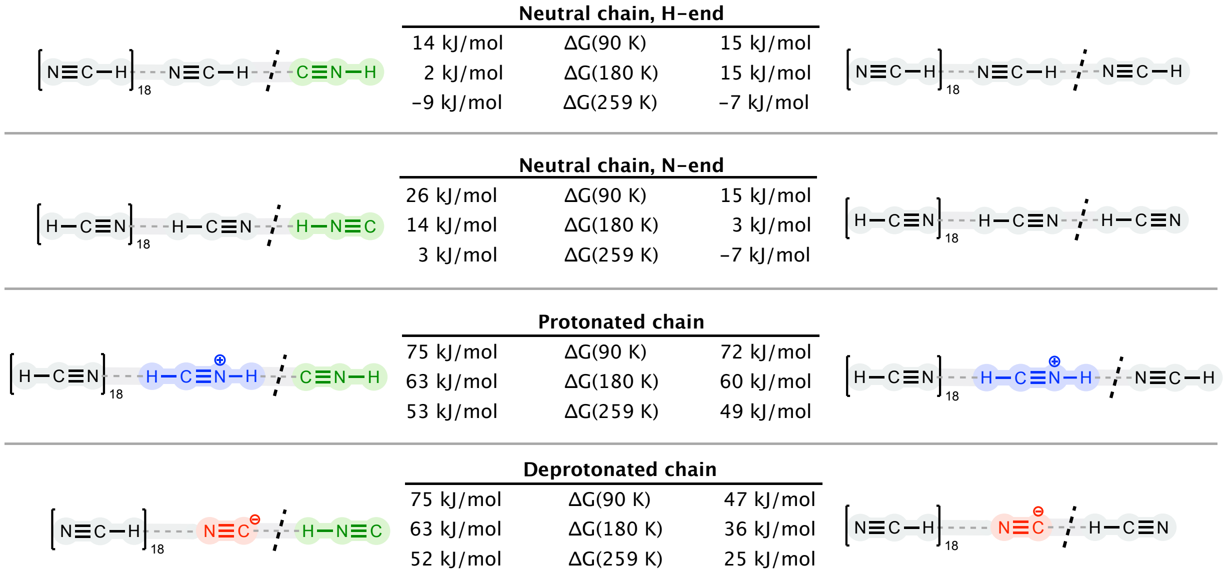

Figure S10: Gas-phase dissociation energies (ΔG) of HNC (left panel) and HCN (right panel) from an HCN chain composed of 19 molecules, at 90 K, at 180 K, and at 259 K. Values for the protonated and the deprotonated chains include proton transfer that forms a terminal HNC (see Figure 5).

4. References

[1] W. J. Dulmage and W. N. Lipscomb, "The Crystal Structures of Hydrogen Cyanide, HCN," *Acta Crystallogr*, vol. 4, p. 334, Jan. 1951.

[2] J. M. Rahm and P. Erhart, "WulffPack: A Python package for Wulff constructions," *The Journal of Open Source Software*, vol. 5, no. 45, 2020, doi: 10.21105/joss.01944.

[3] A. Hjorth Larsen *et al.*, "The atomic simulation environment—a Python library for working with atoms," *Journal of Physics: Condensed Matter*, vol. 29, no. 27, p. 273002, Jun. 2017, doi: 10.1088/1361-648X/AA680E.

[4] P. Virtanen *et al.*, "SciPy 1.0: fundamental algorithms for scientific computing in Python," *Nat Methods*, vol. 17, pp. 261–272, Feb. 2020, doi: 10.1038/s41592-019-0686-2.

[5] S. Shaik, R. Ramanan, D. Danovich, and D. Mandal, "Structure and reactivity/selectivity control by oriented-external electric fields," *Chem Soc Rev*, vol. 47, no. 14, pp. 5125–5145, Jul. 2018, doi: 10.1039/c8cs00354h.

[6] E. P. L. Hunter and S. G. Lias, "Evaluated Gas Phase Basicities and Proton Affinities of Molecules: An Update," *J Phys Chem Ref Data*, vol. 27, no. 3, pp. 413–656, May 1998, doi: 10.1063/1.556018.

[7] S. G. Lias, J. E. Bartmess, J. F. Liebman, J. L. Holmes, R. D. Levin, and W. G. Mallard, "Gas-Phase Ion and Neutral Thermochemistry," *J. Phys. Chem. Ref. Data*, vol. 17, 1988.
