# Supplementary material for: Electric Fields Can Assist Prebiotic Reactivity on Hydrogen Cyanide Surfaces
Source: ACS Cent Sci. 2026 Jan 14;12(1):111–21. doi: 10.1021/acscentsci.5c01497 (PMC12856676; doi:10.1021/acscentsci.5c01497)
Supplement: Supplementary file 2 [file oc5c01497_si_002.pdf]

oc-2025-014973.R1

Name: Peer Review Information for "Electric Fields Can Drive Prebiotic Reactivity on Hydrogen Cyanide Surfaces"

First Round of Reviewer Comments

Reviewer: 1

Comments to the Author

The authors have used computational methods to explore the properties of HCN crystals. Their results explain the high aspect ratios observed for the crystals and they have determined the relative abundances of the different crystal faces. Their results confirm that HCN forms polar crystals with high electric fields at the surfaces. They propose a novel mechanism for the isomerization of HCN to HNC at the surfaces of the HCN crystals. Their mechanism can explain the surprising high HNC/HCN ratio observed in comets and in the atmosphere of Titan. The manuscript is well written, and their conclusions are well supported by their computational results. Their findings are interesting and important. I recommend publication as is.

Reviewer: 2

Comments to the Author

It's an interesting paper - using computational chemistry to examine how a solid HCN crystal can catalyse HCN to HNC -- the context here is mostly in interstellar chemistry - and prebiotic chemistry. But my opinion is that the result doesn't have the sufficient broad impact and major outcomes required for this journal.

The introduction of the paper goes through the instances where HCN chemistry is relevant - in various extraterrestrial situations - but doesn't set up the key broad scientific gap or

problem that this paper then actually addresses. Understanding HCN - HNC isomerisation is an intriguing question -- but the possible process simulated in this paper does not then appear to me to unlock any major outstanding problem in probiotic chemistry or astrochemistry.

Also, the electric field effect doesn't appear to be studied systematically. So I am not sure about the significance - and high electric fields in micro-environments at the surface of crystals would be well known/expected.

So these results appear better suited to a discipline journal. Also, some discussion about any novelty in the computational approach/methods would be helpful.

Reviewer: 3

Comments to the Author

The manuscript argues that oriented local electric fields at step/edge sites on realistic HCN nanocrystals can drive HCN→HNC conversion through two complementary surface cycles (proton addition at N-ends; proton removal/CN^- at H-ends). The crystallography/Wulff construction and chain thermochemistry are well documented: Imm orthorhombic bulk within ~2% of experiment at 0 K; surface energies extrapolated from n-layer slabs to $\gamma(hkl, n\to\infty)$ and interpolated over 2,633 orientations to refine the habit; cooperative chains that raise PA/GB and lower GA, with room-temperature monomer values matching experiment; and dissociation ΔG for terminal HNC/HCN that identifies product egress as the bottleneck. These components are sound and reproducible in principle.

What is missing, however, is the kinetic backbone and field realism at the reactive locus. The SI contains no reaction-path or TS/NEB calculations, and the main text (as provided) does not show barrier-vs-field behaviour or internal fields at steps/terraces; "spontaneous and without barrier" appears only as a qualitative footnote to a chain proton relay. The SI rightly frames terminal HNC detachment as rate-determining with ΔG values too large for

thermal desorption at low T, noting that non-thermal processes may enable propagation—again qualitatively.

1. One field-dependent mechanism with rates: Provide a single, representative reaction path with field orientation control. A relaxed 1D scan plus one TS confirmation (CI-NEB or equivalent) is sufficient—do not over-scope. Report activation free energy for F=0,0.05,0.10 V Å^-1 aligned vs mis-aligned to the reaction axis and convert to Eyring rates at 50–150 K. This can be the N-end proton-relay route that yields terminal HNC (the case presently described as “barrierless”), or the H-end CN^-/deprotonation route—one is enough if done carefully.

2. Quantify internal fields at the site of chemistry: Extract the internal field (or electrostatic potential gradient) at a terrace and at a step/apex from the SCF solution and show an E(z) profile with the field glyph. Add a simple E ~V/a grain-charging cartoon that maps plausible potentials and sizes to the minimum F you actually need; use your Wulff geometry to state a conservative “hot-spot” area fraction (e.g., rim within 1–2 nm of edges/steps). The SI already provides the ingredients (facet energies, interpolation, morphology).

3.Reproducibility and FAIR data: Include full Cartesian coordinates (charge/multiplicity, energies, ZPE/thermals, Nimag) for all chain/cluster minima used, plus slab files (POSCAR/CIF) with field/dipole settings; link the exact Gaussian/ORCA/VASP inputs. The SI notes a dataset; ensure these items are present and cross-referenced from the manuscript.

Reviewer: 4

Comments to the Author

In this work, two possible pathways are discussed to explain the isomerization from HCN to HNC. The novelty of the paper lies in the proposed existence of HCN ice in cryogenic environments, such as Titan’s atmosphere, and tentatively on Triton. For this particular ice, the formation of polar surfaces is studied, as well as their potential catalytic activity due to the generation of strong electric fields.

The modeling of surface catalysis is addressed under Titan's surface conditions (90 K) and also at 259 K. Since HCN ice has been detected in the clouds of Titan's atmosphere, the authors should clarify: what is the temperature in the region where these clouds form? What is the actual temperature range relevant to the proposed model, and to what extent does it align with the catalytic properties of HCN ice as described?

Given that Titan's environment is the main context for the model and that the theoretical results may help explain the complex chemistry occurring on this moon, the general introduction should be more clearly focused in this direction. The use of the term astrochemical environment may be confusing for the general astrobiology reader, such as myself, since it is typically used to refer to the interstellar medium (ISM), where HCN and HNC are expected to exist in the gas phase. I would therefore recommend rewriting the introduction to simplify and clarify the context, framing the study within the planetary scenario of Titan as a representative cryogenic environment in the Solar System.

Titan is a fascinating object of study in its own right, as is the intriguing chemistry of HCN. Regarding the section titled Speculation on astrochemical relevance, I believe the main focus should remain on Titan. While the hypothesis concerning the HCN/HNC ratio in comets is interesting, it remains highly speculative, especially considering that, as the authors note, HCN ice has not been detected in comets.

In this sense, the paper should be more nuanced: the authors could clarify that the modeling is primarily aimed at understanding possible isomerization reactions in cold environments, with a first approximation focused on Titan's conditions. These results could then potentially be extrapolated to other scenarios, such as comets, but this should be presented as a secondary implication rather than a central theme.

Reviewer: 5

Comments to the Author

The manuscript presents a study on HCN crystals and their relevance to astrochemical environments, with a focus on low-temperature (~170 K) phases. The calculation of HCN

surface energies and the investigation of their potential role in catalyzing HNC formation is an interesting aspect of the work.

I have two major concerns:

1. The manuscript discusses two astrochemical environments: comets and Titan's atmosphere. While comets are a reasonable connection, Titan's atmosphere is more complex. In particular, processes such as atmospheric photochemistry, aerosols, stellar high-energy radiation and particle fluxes, and cosmic rays could contribute to the observed HNC abundances. It is not clear how the surface-catalyzed HNC formation mechanism fits with these atmospheric observations. This connection is currently missing and should be addressed.

2. It is unclear whether this is the first Wulff construction performed for HCN or if similar work has been done previously. The manuscript should clarify the novelty of these results. If prior studies exist, a comparison with the current analysis would be helpful. If this is the first Wulff construction for HCN, this should be explicitly stated.

I also have a few minor comments related to the points above:

Page 1, L46: even a rocky exoplanet.

=> there has been no confirmed detection of an atmosphere on a rocky exoplanet. Also, most exoplanets observed today have temperatures much higher than 170 K. A sentence or two on the relevance of this study for these hotter environments would be useful.

Page 2, L52-56: Discussion on high HNC/HCN ratios

=> The manuscript doesn't discuss the potential impact of cosmic rays. Cosmic rays can penetrate deeper in the atmosphere and drive ionization reactions. A sentence or two on whether that could impact the HNC/HCN ratios observed in Titan would be useful.

Page 4, L7-10, column 2: These results are in agreement with previous studies .....

=> The manuscript mentions agreement with a prior study but doesn't mention how this study differs from citation 88. The text could also highlight the new insights gained from this work that were not available in the past.

Page 5, L8-10: Titan’s upper atmosphere

=> It is not clear what the manuscript means by upper atmosphere of Titan. Exact pressure ranges would be useful here. For astrophysicists the upper atmosphere is pure gas and no solid particles. The upper atmosphere is also much hotter than the lower atmosphere as it absorbs high energy stellar radiation. Under such environments photochemistry, photoionization and escaping gas would impact the HNC/HCN ratio. I suspect the manuscript refers to the middle atmosphere, i.e. below the thermosphere.

Reviewer: 6

Comments to the Author

In this manuscript, Cappelletti, Sandstrom, and Rahm use density functional theory-based calculations to investigate the potential for interfacial electric fields that arise from polar HCN surfaces to drive the isomerization of HCN to HNC. First, they use the Wulff construction combined with the calculation of surface energies to predict the morphology of the HCN crystal and find good agreement with the needle-like structures found in experiments. They also predict that, while polar surfaces are not the majority, they do make up a finite fraction of the crystal surface, enabling them to act as catalytic surfaces. The manuscript then examines HCN isomerization on N or H terminated polar surfaces, finding rapid proton transfer and therefore isomerization to HNC, suggesting that these polar surfaces could indeed facilitate chemistry through the large electric fields produced by cooperative effects in the crystal.

The methodology is sound, and the results are impactful. The manuscript does a nice job discussing the potential impact of the findings on astrochemistry and astrobiology/prebiotic chemistry, especially relevant to Saturn’s moon Titan and chemistry on comets. I recommend publication as is.

Author's Response to Peer Review Comments:

Chalmers University of Technology Department of Chemistry and Chemical Engineering
Division of Chemistry and Biochemistry

November 04, 2025

ACS Central Science

Dear Editor,

We are pleased to submit a revision of our manuscript entitled "*Electric Fields Can Drive Prebiotic Reactivity on Hydrogen Cyanide Surfaces*" for your consideration for publication in ACS Central Science.

We would like to thank all reviewers for their insightful feedback. We have done our utmost to oblige all suggestions and requests in changes and additions to the manuscript and the supporting information. All changes are marked in separate docx files, and most are shown below in our reviewer response.

Thank you for considering our revised manuscript.

Sincerely,

Martin Rahm, Chalmers University of Technology On behalf of all authors

DEPARTMENT OF CHEMISTRY AND CHEMICAL ENGINEERING

Division of Chemistry and Biochemistry
Chalmers University of Technology
SE-412 96 Gothenburg, Sweden
Visiting address: Kemigården 4, forskarhus 1, room 5019
Phone: +46 31-772 30 50
E-mail: martin.rahm@chalmers.se Web: www.rahmlab.com
Chalmers tekniska högskola AB
Reg.No: 556479-5598 VAT No: SE556479559801

Replies to Comments by Reviewer 1

R1: "The authors have used computational methods to explore the properties of HCN crystals. Their results explain the high aspect ratios observed for the crystals and they have determined the relative abundances of the different crystal faces. Their results confirm that HCN forms polar crystals with high electric fields at the surfaces. They propose a novel mechanism for the isomerization of HCN to HNC at the surfaces of the HCN crystals. Their mechanism can explain the surprising high HNC/HCN ratio observed in comets and in the atmosphere of Titan. The manuscript is well written, and their conclusions are well supported by their computational results. Their findings are interesting and important. I recommend publication as is." *Authors reply:* Thank you very much for seeing the value of our work.

Replies to Comments by Reviewer 2

R2: "It's an interesting paper - using computational chemistry to examine how a solid HCN crystal can catalyse HCN to HNC -- the context here is mostly in interstellar chemistry - and prebiotic chemistry. But my opinion is that the result doesn't have the sufficient broad impact and major outcomes required for this journal.

The introduction of the paper goes through the instances where HCN chemistry is relevant - in various extraterrestrial situations - but doesn't set up the key broad scientific gap or problem that this paper then actually addresses. Understanding HCN - HNC isomerisation is an intriguing question -- but the possible process simulated in this paper does not then appear to me to unlock any major outstanding problem in probiotic chemistry or astrochemistry."

Authors reply: Thank you for your comment. In or revision we have focused our work more on HCN's role in Titan's settings, removed the mention of interstellar medium (ISM), and lessened the focus on comets. We also state knowledge gap and problems more explicitly, for example by writing:

> "Deciphering the structure, distribution and properties of HCN ice on Titan is therefore essential for understanding both chemical and geological evolution of this world.[24] There is growing evidence that ethane and methane, the main components of Titan's lakes and seas, can intercalate into the HCN crystal lattice, forming co-crystals that could take the role of cryogenic minerals.[30] Whether solid-state HCN is chemically active in this setting – and to what degree its higher energy isomer HNC can form from it – remains an open question that we will return to discuss."

And

> "In what follows, we predict energies of HCN surfaces and show them to be commensurate with observed crystal morphology. We also propose a family of novel HCN↔HNC isomerization mechanisms, enabled by the ionization of solid HCN polar surfaces, which may contribute to explaining the HNC abundance anomaly in cold HCN-rich environments, such as Titan."

And

> "The potential for an HCN crystal to drive chemical reactions arguably depends on the fractional area occupied by polar surfaces, where electric fields are strongest. Estimating the prevalence of such surfaces reduces to the question of estimating the shape of HCN nanocrystals. However, as far as we can determine, the equilibrium crystal shape of pure HCN has not been reported, experimentally or computationally. To do so, we…"

And

> "We think that the mechanisms outlined in Figure 5 offer a plausible explanation for the observed abundance anomaly of HNC in Titan's atmosphere, and for how solid-phase HCN may participate in dynamic, surface-driven transformations at low temperatures. While these mechanisms are but models, they highlight how surface-catalyzed chemistry may be particularly relevant in Titan's complex chemical environment."

R2: "Also, the electric field effect doesn't appear to be studied systematically. So I am not sure about the significance - and high electric fields in micro-environments at the surface of crystals would be well known/expected."

Authors reply: While it is true that sizeable electric fields can be expected on the surfaces of some polar crystals, we disagree that the

magnitude of their intensity is well known. The HCN crystal represents an extreme situation. In our revision we have studied the field effects more systematically and added the section “Energetics of the isomerization mechanisms” to the SI, showing the effect of the electric fields on the HCN to HNC isomerization energy and barrier:

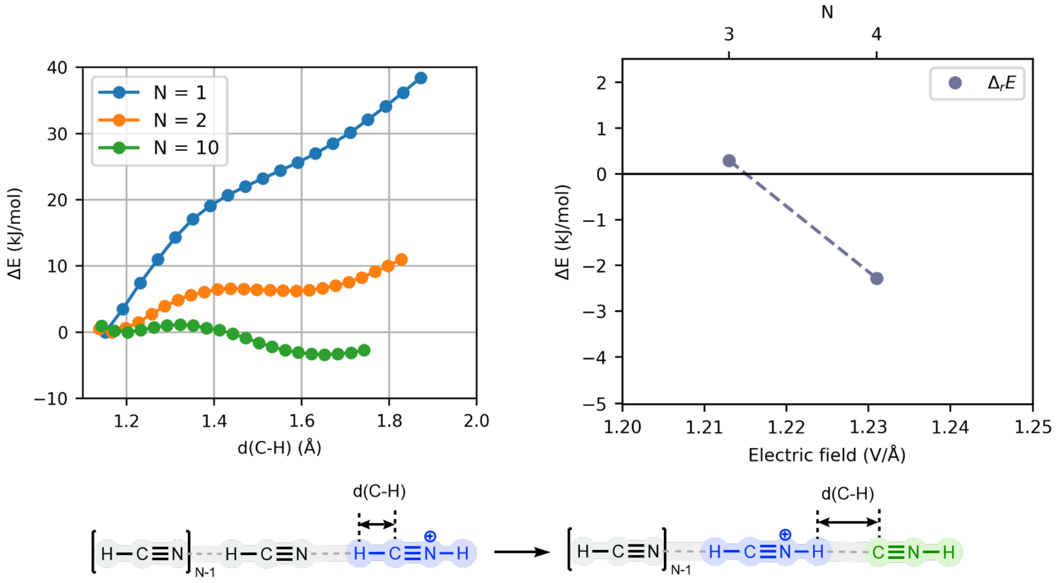

Figure S8. Energetics of the cationic HCN to HNC isomerization mechanism. Left plot: Relaxed scan of the proton transfer, isolated (N=1, in blue), or catalyzed by either a single HCN (N=2, orange), or a 9-HCN-unit chain (N=10, green). Plotted are DLPNO-CCSD(T)/aug-cc-pVTZ//B3LYP-D3(BJ)/ 6-311++G(d,p) electronic energies against C-H distance, as shown in the bottom scheme. Right plot: Reaction energy ($\Delta_r E$) (DLPNOCCSD(T)/aug-cc-pVTZ//B3LYP-D3(BJ)/ 6-311++G(d,p) electronic energies) of the mechanism against the electric field at 1.9 Å from the N-end of the linear chain. The length of the chain (N) is also shown in the upper horizontal axis. For N<3, the product state is not a minimum in the DFT PES; conversely, for N>4, the reactant state is not a minimum in the DFT PES. Transition states could not be identified for any of these reactions.

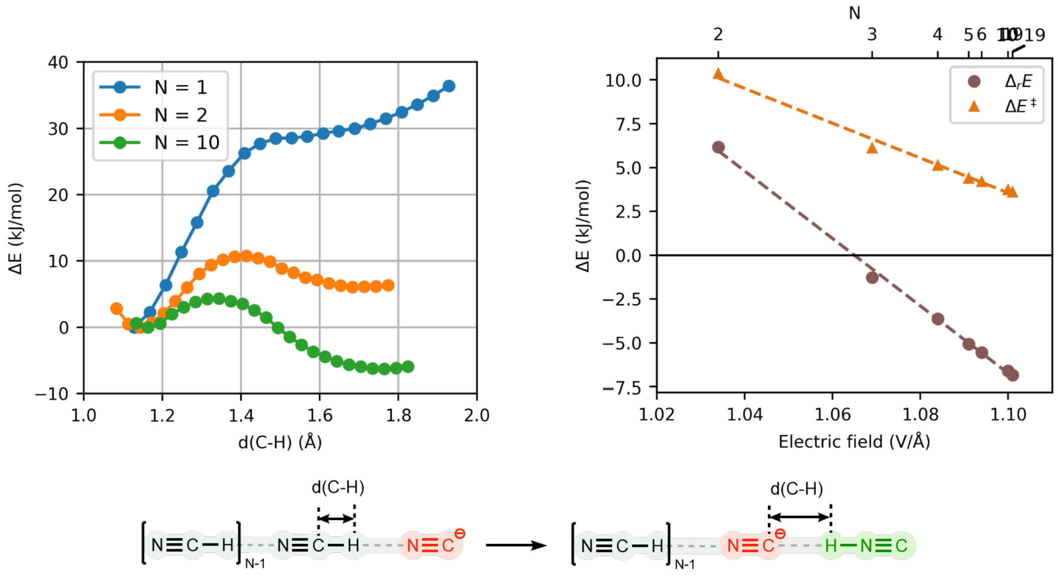

Figure S9. Energetics of the anionic HCN to HNC isomerization mechanism. Left plot: Relaxed scan of the proton transfer, isolated (N=1, in blue), or catalyzed by either a single HNC (N=2, orange) or a 9-HCN-unit chain (N=10, green). Plotted are DLPNO-CCSD(T)/aug-cc-pVTZ//B3LYP-D3(BJ)/ 6-311++G(d,p) electronic energies against C-H distance, as shown in the bottom scheme. Right plot: Reaction energy (Δ_rE) and reaction barrier ($\Delta E^{\ddagger}$) (DLPNO-CCSD(T)/aug-cc-pVTZ//B3LYP-D3(BJ)/ 6-311++G(d,p) electronic energies) of the mechanism against the electric field at 1.9 Å from the H-end of the linear chain. The length of the chain (N) is also shown in the upper horizontal axis. The uncatalyzed isomerization mechanism is unfavored and the product is not a minimum in the potential energy surface. The isomerization becomes favored as N≥3. Both the reaction energy and the barrier decrease linearly with the intensity of the electric field.

And the following to the main text:

> "It is notable that the HCN surface not only catalyzes HNC formation, but also switches the thermodynamic preference relative to the non-catalyzed the gas-phase mechanism: whereas gas-phase proton transfer between $HCNH^+$ and HNC to yield HCN and $HCNH^+$ is barrierless and exergonic, HNC formation becomes favored on the protonated surface once the chain is composed of four or more units (Figure S8). This result is in line with previous computational studies focused on ionized (open-shell) clusters,[86] and, in our calculations, the energetics of the process correlates with the intrinsic surface electric field (Figure S8). We therefore assert that such surface-catalyzed HCN↔HNC isomerization is electrostatically induced."

and

> "Similarly to the cationic mechanism, this process appears to be electrostatically induced, as both the reaction energy and the reaction barrier linearly correlate with the electric field at the H-end of the chain (Figure S9)."

Moreover, we have computed the electric field from the (001) and (001") surfaces, and added the following Figure to the SI:

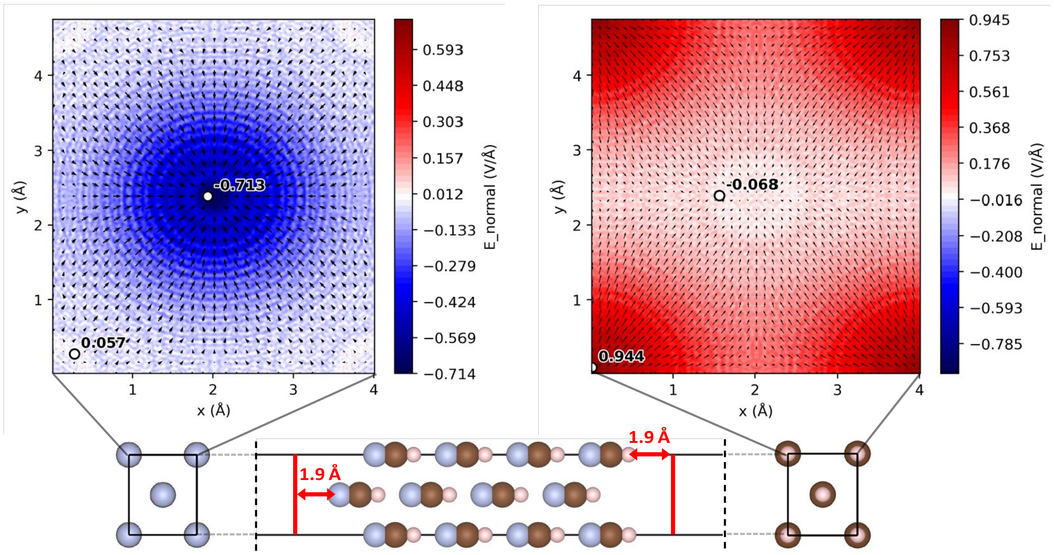

Figure S7. 2D plots of the electric field intensity at 1.9 Å from the N-end (left) and from the H-end (right) surface of a 4-HCN-unit (001) slab. The contour represents the out-of-plane (normal) component of the electric field, and overlayed is a quiver plot of the in-plane projected electric field vectors. Positive values (in red) indicate a normal component exiting the plane, while negative values (in blue) indicate a normal component entering the plane. Labeled data points show the minimum and the maximum value of the normal component. The field appears somewhat dampened compared to the linear chain, especially at the N-end.

Which we also mention in the main text:

> "We motivate the use of a chain model by the higher level of theory it permits compared to periodic slab models of the {001} surface (Figure S7). Slab models appear to predict slightly lower field strengths (e.g., 1.20 →0.95 V/Å for an H-terminated surface, Figure S7) compared to a single chainmodel but suffer noise and method sensitivity. We will return to analyze the electronic structure of HCN crystal surfaces responsible for such differences in future work.
>
> Regardless of the model choice, the predicted electric fields on polar surfaces are large. Linear association of HCN is clearly able to generate field strengths of the same order of magnitude as proteins and scanning tunneling microscopy tips (Figure S6), both known to facilitate chemical transformations.[101–103] For example, a field of ~0.9 V/Å is able to induce the dissociation of weakly bonded molecules, e.g., in Na_2, Li_2.[56]"

R2: "So these results appear better suited to a discipline journal. Also, some discussion about any novelty in the computational approach/methods would be helpful."

Authors reply: We do not claim to present a novel computational approach: rather we use well established state-of-the-art tools and methods so to be certain of their validity. Our focus is on the scientific question's surrounding HCN's chemistry in different environments. To

better emphasize the novelty of our work we now state early in the Results and Discussion section that:

> "However, as far as we can determine, the equilibrium crystal shape of pure HCN has not been reported, experimentally or computationally. To do so, we.."

Replies to Comments by Reviewer 3

R3: "The manuscript argues that oriented local electric fields at step/edge sites on realistic HCN nanocrystals can drive HCN→HNC conversion through two complementary surface cycles (proton addition at N-ends; proton removal/CN^- at H-ends). The crystallography/Wulff construction and chain thermochemistry are well documented: Imm orthorhombic bulk within ~2% of experiment at 0 K; surface energies extrapolated from n-layer slabs to γ(hkl,n→∞) and interpolated over 2,633 orientations to refine the habit; cooperative chains that raise PA/GB and lower GA, with room-temperature monomer values matching experiment; and dissociation ΔG for terminal HNC/HCN that identifies product egress as the bottleneck. These components are sound and reproducible in principle.

What is missing, however, is the kinetic backbone and field realism at the reactive locus. The SI contains no reaction-path or TS/NEB calculations, and the main text (as provided) does not show barrier-vs-field behaviour or internal fields at steps/terraces; "spontaneous and without barrier" appears only as a qualitative footnote to a chain proton relay. The SI rightly frames terminal HNC detachment as rate-determining with ΔG values too large for thermal desorption at low T, noting that non-thermal processes may enable propagation—again qualitatively.

1. One field-dependent mechanism with rates: Provide a single, representative reaction path with field orientation control. A relaxed 1D scan plus one TS confirmation (CI-NEB or equivalent) is sufficient—do not over-scope. Report activation free energy for F=0,0.05,0.10 V Å^1 aligned vs mis-aligned to the reaction axis and convert to Eyring rates at 50–150 K. This can be the N-end proton-relay route that yields terminal HNC (the case presently described as

"barrierless"), or the H-end CN^-/deprotonation route—one is enough if done carefully.

Authors reply: Thank you for the interesting comment. A quantitative estimation of the proton transfer mechanism, and a more systematic study of the electric field effect was missing in our original submission. We have added Figures S8 and S9, shown above, which contain the results of 1D relaxed scans of both mechanisms, isolated and catalyzed by a linear HCN chain. The figures also present the reaction energies and barriers (where existing) of the processes versus the electric field at 1.9 Å from the linear chains. A misaligned external electric field would be unphysical, as the electric fields are inherent to the linear chain/polar surfaces, and therefore we did not include such testing. Regarding rates, we state:

> "We compute the reaction energy barrier for this step to be very small, ~4 kJ/mol, which corresponds to a first order reaction timescale in the order of picoseconds at 90 K."

Given that the proton transfer step is not rate determining in any situation, it is not meaningful to attempt prediction of more precise numbers here. It is clearly the desorption of HNC which is rate-limiting. To attain accurate rates for the (again non-rate-determining) H-transfer steps, one would need to account for tunneling, and expand the study substantially well outside our scope. We believe the following semi-quantitative statements are honest representations of our model's accuracy. Providing rates from our calculations would communicate a level of accuracy not present (or even possible given the poor constraints on the Titan's complex atmosphere):

> "The rate-determining step in this mechanism is the dissociation of HNC, for which we estimate an Gibbs energy barrier of approximately 75, 62, and 52 kJ/mol at 90, 180 and 259 K, respectively (Figure S10). In other words, while this barrier is far too large to allow for thermal desorption of HNC on the surface of Titan, it does permit reactions on the timescale of days at ~180 K, close to the temperature where HCN ice is believed to form and persist on Titan. At higher temperatures near the melting point (259 K), the barrier is low enough to allow isomerization on the timescale of milliseconds. These estimates were obtained using the Eyring equation, assuming first-order reaction kinetics."

R3: "2. Quantify internal fields at the site of chemistry: Extract the internal field (or electrostatic potential gradient) at a terrace and at a

step/apex from the SCF solution and show an E(z) profile with the field glyph. Add a simple E ~V/a grain-charging cartoon that maps plausible potentials and sizes to the minimum F you actually need; use your Wulff geometry to state a conservative "hot-spot" area fraction (e.g., rim within 1–2 nm of edges/steps). The SI already provides the ingredients (facet energies, interpolation, morphology)."

Authors reply: Thank you. We believe this comment to be the result of a misunderstanding. To quantum chemically compute electric fields for a large-scale Wulff construction containing millions of atoms is unfeasible. In our revision we have computed the electric field at 1.9 Å from the surface of a small (4-HCN-unit thick) (001) slab (Figure S7, reproduced above). While we noticed a small discrepancy of the electric field compared to the linear chain model, we nonetheless use the latter as to allows the employment of a much higher level of theory (DLPNOCCSD(T) //B3LYP-D3(BJ) compared to PBE-D3(BJ)). To clarify, we now state:

> "We motivate the use of a chain model by the higher level of theory it permits compared to periodic slab models of the {001} surface (Figure S7). Slab models appear to predict slightly lower field strengths (e.g., 1.20 →0.95 V/Å for an H-terminated surface, Figure S7) compared to a single chainmodel but suffer noise and method sensitivity. We will return to analyze the electronic structure of HCN crystal surfaces responsible for such differences in future work.
>
> Regardless of the model choice, the predicted electric fields on polar surfaces are large. Linear association of HCN is clearly able to generate field strengths of the same order of magnitude as proteins and scanning tunneling microscopy tips (Figure S6), both known to facilitate chemical transformations.[101–103] For example, a field of ~0.9 V/Å is able to induce the dissociation of weakly bonded molecules, e.g., in Na_2, Li_2.[56]"

R3: "3.Reproducibility and FAIR data: Include full Cartesian coordinates (charge/multiplicity, energies, ZPE/thermals, Nimag) for all chain/cluster minima used, plus slab files (POSCAR/CIF) with field/dipole settings; link the exact Gaussian/ORCA/VASP inputs. The SI notes a dataset; ensure these items are present and cross-referenced from the manuscript."

Authors reply: This data was available for review in our original submission. All relevant files have been uploaded to the Swedish National Database (SND), and the link is provided in SI. We have further revised and updated these files for this revision.

Replies to Comments by Reviewer 4

R4: "In this work, two possible pathways are discussed to explain the isomerization from HCN to HNC. The novelty of the paper lies in the proposed existence of HCN ice in cryogenic environments, such as Titan's atmosphere, and tentatively on Triton. For this particular ice, the formation of polar surfaces is studied, as well as their potential catalytic activity due to the generation of strong electric fields.

The modeling of surface catalysis is addressed under Titan's surface conditions (90 K) and also at 259 K. Since HCN ice has been detected in the clouds of Titan's atmosphere, the authors should clarify: what is the temperature in the region where these clouds form? What is the actual temperature range relevant to the proposed model, and to what extent does it align with the catalytic properties of HCN ice as described?

Authors reply: Thank you for these insightful comments. In response, we have re-focused our work more directly on the Titan environment and clarify in the introduction section:

> "While HCN ice has yet to be confirmed in comets, it has been tentatively detected in the atmosphere of Neptune's moon Triton,[21] and observed in large amounts in the atmosphere of Saturn's moon Titan,[22] an environment we focus on.
>
> HCN is one of the major products of Titan's upper atmospheric chemistry.[23,24] After formation, it drifts downward and condenses together with other reaction products into aerosols that feed Titan's complex yellow haze. Condensation of HCN ice can occur at altitudes below approximately 300 km,[22] depending on temperature.[25] At the Huygens site, modeling indicates that HCN begins to condense near ~75 km, with pure HCN cloud particles extending to ~30 km.[26] A south-polar cloud incorporating crystalline HCN was observed at ~300 km in 2012, later descending toward ~200 km as the season progressed, implying local temperatures near the HCN frost point (~125 K) at those altitudes.[27] Over time, an estimated 2 mm per Myr of HCN ice (or HCN-equivalences in reaction products) is believed to accumulate on Titan's ~94 K surface.[28,29] Deciphering the structure, distribution and properties of HCN ice on Titan is therefore essential for understanding both chemical and geological evolution of this world.[24] There is growing evidence that ethane and methane, the main components of Titan's lakes and seas, can intercalate into the HCN crystal lattice, forming co-crystals that could take the role of cryogenic minerals.[30] Whether solid-state HCN is chemically active in this setting – and to what degree its higher energy isomer HNC can form from it – remains an open question that we will return to discuss."

As well as connecting back to the Titan environment on several other instances in the main text (most omitted here), for example:

"In other words, while this barrier is far too large to allow for thermal desorption of HNC on the surface of Titan, it does permit reactions on the timescale of days at ~180 K, close to the temperature where HCN ice is believed to form and persist on Titan. At higher temperatures near the melting point (259 K), the barrier is low enough to allow isomerization on the timescale of milliseconds. These estimates were obtained using the Eyring equation, assuming first-order reaction kinetics."

R4: "Given that Titan's environment is the main context for the model and that the theoretical results may help explain the complex chemistry occurring on this moon, the general introduction should be more clearly focused in this direction. The use of the term astrochemical environment may be confusing for the general astrobiology reader, such as myself, since it is typically used to refer to the interstellar medium (ISM), where HCN and HNC are expected to exist in the gas phase. I would therefore recommend rewriting the introduction to simplify and clarify the context, framing the study within the planetary scenario of Titan as a representative cryogenic environment in the Solar System.

Titan is a fascinating object of study in its own right, as is the intriguing chemistry of HCN. Regarding the section titled Speculation on astrochemical relevance, I believe the main focus should remain on Titan. While the hypothesis concerning the HCN/HNC ratio in comets is interesting, it remains highly speculative, especially considering that, as the authors note, HCN ice has not been detected in comets.

In this sense, the paper should be more nuanced: the authors could clarify that the modeling is primarily aimed at understanding possible isomerization reactions in cold environments, with a first approximation focused on Titan's conditions. These results could then potentially be extrapolated to other scenarios, such as comets, but this should be presented as a secondary implication rather than a central theme."

Authors reply: Thank you for these remarks, which we agree with. We have now reworked the text to shift our focus away from the ISM to Titan, and clarified our main focus (see previous answers). We have left a discussion on comets in the section labeled "Speculations on Astrochemical Relevance", while also clarifying that it is more a speculative hypothesis.

Replies to Comments by Reviewer 5

R5: “The manuscript presents a study on HCN crystals and their relevance to astrochemical environments, with a focus on low-temperature (~170 K) phases. The calculation of HCN surface energies and the investigation of their potential role in catalyzing HNC formation is an interesting aspect of the work.

I have two major concerns:

1. The manuscript discusses two astrochemical environments: comets and Titan’s atmosphere. While comets are a reasonable connection, Titan’s atmosphere is more complex. In particular, processes such as atmospheric photochemistry, aerosols, stellar high-energy radiation and particle fluxes, and cosmic rays could contribute to the observed HNC abundances. It is not clear how the surface-catalyzed HNC formation mechanism fits with these atmospheric observations. This connection is currently missing and should be addressed.

Authors reply: Thank you for this comment, which echoes some of the other reviewers. Besides our changes outlined above, we now more clearly acknowledge that Titan’s atmospheric chemistry is complex in the section on “Speculation on Astrochemical Relevance”. What we advance is a hypothesis on where and how our proposed mechanisms may take place:

> “We think that the mechanisms outlined in Figure 5 offer a plausible explanation for the observed abundance anomaly of HNC in Titan’s atmosphere, and for how solid-phase HCN may participate in dynamic, surface-driven transformations at low temperatures. While these mechanisms are but models, they highlight how surface-catalyzed chemistry may be particularly relevant in Titan’s complex chemical environment. HCN is one of the major products of the atmospheric photochemistry of Titan,[23] and micron-sized HCN ice particles have been observed at high altitude near its southern pole.[22] This environment is bathed in ionizing radiation from the sun and Saturn’s magnetosphere, and the atmospheric layers below are rich in photochemically generated ions, including $HCNH^+$, that could facilitate HCN → HNC isomerization via the mechanisms we have described. Once formed, HNC may remain bound to the surface or be released into the gas phase when the particles are lofted into warmer atmospheric layers or exposed to UV photons or charged particle irradiation. Meanwhile, new HNC is regenerated that could either become trapped in the lattice or continue the release. This process would provide a dynamic route for modulating the gas-phase nitrile composition without requiring bulk sublimation of HCN ice. It could also contribute to the vertical redistribution and chemical evolution of nitriles in Titan’s stratified atmosphere.”

R5: "2. It is unclear whether this is the first Wulff construction performed for HCN or if similar work has been done previously. The manuscript should clarify the novelty of these results. If prior studies exist, a comparison with the current analysis would be helpful. If this is the first Wulff construction for HCN, this should be explicitly stated."

Authors reply: This is indeed the first time that Wulff construction is applied to HCN, and the first time a study on HCN's crystal shape is reported. To clarify this, we now begin the Results and Discussion section as follows:

"RESULTS AND DISCUSSION

HCN Surface Energies and Crystal Morphology

The potential for an HCN crystal to drive chemical reactions arguably depends on the fractional area occupied by polar surfaces, where electric fields are strongest. Estimating the prevalence of such surfaces reduces to the question of estimating the shape of HCN nanocrystals. However, as far as we can determine, the equilibrium crystal shape of pure HCN has not been reported, experimentally or computationally. To do so, we rely on Wulff's theorem, which states that the equilibrium shape of a single crystal, the so-called Wulff construction, is that which minimizes the total surface energy.[94,95]"

R5: "I also have a few minor comments related to the points above:

Page 1, L46: even a rocky exoplanet.

=> there has been no confirmed detection of an atmosphere on a rocky exoplanet." *Authors reply:* Thank you for spotting this! Now removed.

R5: "Also, most exoplanets observed today have temperatures much higher than 170 K. A sentence or two on the relevance of this study for these hotter environments would be useful." *Authors reply:* A good point. We now state the following in the first paragraph of the Results and Discussion section:

"Surface energies (γ) are here calculated by means of periodic Density Functional Theory (DFT), the details of which are outlined in the Methods section. Because our focus

is on cryogenic environments, we limit our study to the orthorhombic *Imm2* phase. Given that the high-temperature tetragonal *I4mm* phase retains the same polar chain topology, we expect our qualitative conclusions to carry over to the 259 K melting point, although quantitative surface energies will be phase dependent."

R5: "Page 2, L52-56: Discussion on high HNC/HCN ratios=> The manuscript doesn't discuss the potential impact of cosmic rays. Cosmic rays can penetrate deeper in the atmosphere and drive ionization reactions. A sentence or two on whether that could impact the HNC/HCN ratios observed in Titan would be useful."

Authors reply: Thank you for this suggestion. As noted also above, we have significantly reworked the introduction to focus more on Titan, and we now state the following in the introduction relating to cosmic rays specifically:

"On Titan specifically, photochemical models indicate that most HNC forms in the ionosphere,[90] with one important source being $HCNH^+$,[28,29,91] which forms through protonation of HCN. Cosmic rays also penetrate Titan's dense atmosphere and peaks in ionization at ~65 km,[92] where it can drive chemical transformations.

Besides the unexplained high HNC/HCN ratio in Titan's atmosphere, a second potential anomaly is the sharp decline of HNC with altitude.[93] Although HNC→ HCN is thermodynamically favored, known neutral re-isomerization barriers are prohibitively high at Titan temperatures, making chemical reactivity, cosmic rays or photolysis more likely explanations."

R5: "Page 4, L7-10, column 2: These results are in agreement with previous studies .....

=> The manuscript mentions agreement with a prior study but doesn't mention how this study differs from citation 88. The text could also highlight the new insights gained from this work that were not available in the past."

Authors reply: Reference 88 (now 86) mainly focuses on ionization of small (2-6 units) HCN clusters, either by adding or removing an electron from the system. while our main focus is closed-shell ion chemistry, while also studying the electric field effect. To clarify, we have included the following paragraph:

"It is notable that the HCN surface not only catalyzes HNC formation, but also switches the thermodynamic preference relative to the non-catalyzed the gas-phase mechanism: whereas gas-phase proton transfer between $HCNH^+$ and HNC to yield HCN and $HCNH^+$

is barrierless and exergonic, HNC formation becomes favored on the protonated surface once the chain is composed of four or more units (Figure S8). This result is in line with previous computational studies focused on ionized (open-shell) clusters,[86] and, in our calculations, the energetics of the process correlates with the intrinsic surface electric field (Figure S8). We therefore assert that such surface-catalyzed HCN↔HNC isomerization is electrostatically induced."

R5: "Page 5, L8-10: Titan's upper atmosphere

=> It is not clear what the manuscript means by upper atmosphere of Titan. Exact pressure ranges would be useful here. For astrophysicists the upper atmosphere is pure gas and no solid particles. The upper atmosphere is also much hotter than the lower atmosphere as it absorbs high energy stellar radiation. Under such environments photochemistry, photoionization and escaping gas would impact the HNC/HCN ratio. I suspect the manuscript refers to the middle atmosphere, i.e. below the thermosphere."

Authors reply: Thank you for this comment. We have tried to be clearer regarding the altitudes where HCN can be found on Titan (see our responses above). We now mention that while HCN forms predominantly in the upper atmosphere, HCN ice can only be found in the lower atmosphere (0-300 km).

Replies to Comments by Reviewer 6

R6: "In this manuscript, Cappelletti, Sandstrom, and Rahm use density functional theory-based calculations to investigate the potential for interfacial electric fields that arise from polar HCN surfaces to drive the isomerization of HCN to HNC. First, they use the Wulff construction combined with the calculation of surface energies to predict the morphology of the HCN crystal and find good agreement with the needle-like structures found in experiments. They also predict that, while polar surfaces are not the majority, they do make up a finite fraction of the crystal surface, enabling them to act as catalytic surfaces. The manuscript then examines HCN isomerization on N or H terminated polar surfaces, finding rapid proton transfer and therefore isomerization to HNC, suggesting that these polar surfaces could indeed facilitate chemistry through the large electric fields produced by cooperative effects in the crystal.

The methodology is sound, and the results are impactful. The manuscript does a nice job discussing the potential impact of the findings on astrochemistry and astrobiology/prebiotic chemistry, especially relevant to Saturn's moon Titan and chemistry on comets. I recommend publication as is."

Authors reply: Thank you very much for seeing the value of our work.

oc-2025-014973.R2

Name: Peer Review Information for "Electric Fields Can Assist Prebiotic Reactivity on Hydrogen Cyanide Surfaces"

Second Round of Reviewer Comments

Reviewer: 3

Comments to the Author

Remaining revision points:

(1) Assisted egress and HNC release. The authors now provide detachment free energies and estimate Eyring timescales. They correctly conclude that thermal release is negligible under Titan surface temperatures but becomes feasible at warmer layers. This is a meaningful improvement. However, they still do not include even a simple numerical order-of-magnitude estimate for non-thermal release pathways such as CR-UV photodesorption, solar-wind or GCR processing, or chemical kick-out. As a result, the plausibility of net HNC export from cold grains remains only qualitatively supported.

(2) Hybrid functional robustness check. The revised methods are much clearer overall, but the requested single hybrid-functional spot check under field for the key step is still missing. This should be added to bound self-interaction error and confirm that the field trends are not artefacts of the chosen GGA.

(3) Astrochemical bridge. The new paragraph linking the mechanism to Titan and comets is well written and strengthens the relevance. Yet it remains a narrative argument. A short scaling or box-model estimate, even three or four lines, would greatly improve the quantitative credibility of the proposed contribution to observed HCN/HNC behaviour.

(4) Language and scope. The manuscript continues to use strong "drive/catalyse" framing without the quantitative rate enhancement needed to fully justify it. In addition, the term "cosmic wind" is non-standard. It should be replaced with precise terminology such as "solar-wind ions" for Titan and comets, or "galactic cosmic rays and secondary electrons" for dense-cloud environments. Unless the authors add quantitative rate support, the strongest claims in the title and abstract should be softened to "field-assisted pathways that may contribute under relevant conditions.

Reviewer: 2

Comments to the Author

The authors have done a very good job and address my comments and critiques.

And on that baisis, I am comfortable to recommend publicaiton.

Author's Response to Peer Review Comments:

Chalmers University of Technology Department of Chemistry and Chemical Engineering
Division of Chemistry and Biochemistry

December 04, 2025

Senior Editor, *ACS Central Science*

Dear Editor,

We are pleased to submit our second revision of our manuscript, now titled "*Electric Fields Can Assist Prebiotic Reactivity on Hydrogen Cyanide Surfaces*" for your consideration for publication in ACS Central Science. We think we have addressed all remaining points by Reviewer 3. All changes are marked A track changes in docx files, and most are shown in red in our reviewer response below.

Thank you for considering our revised manuscript.

Sincerely,
Martin Rahm, Chalmers University of Technology
On behalf of all authors

DEPARTMENT OF CHEMISTRY AND CHEMICAL ENGINEERING

Division of Chemistry and Biochemistry
Chalmers University of Technology
SE-412 96 Gothenburg, Sweden
Visiting address: Kemigården 4, forskarhus 1, room 5019
Phone: +46 31-772 30 50
E-mail: martin.rahm@chalmers.se Web: www.rahmlab.com
Chalmers tekniska högskola AB
Reg.No: 556479-5598 VAT No: SE556479559801

Replies to Comments by Reviewer 2

R2: " The authors have done a very good job and address my comments and critiques. And on that basis, I am comfortable to recommend publication."

Authors reply: Thank you very much for your comments, and for seeing the value of this work.

Replies to Comments by Reviewer 3

R3: "Remaining revision points:

(1) Assisted egress and HNC release. The authors now provide detachment free energies and estimate Eyring timescales. They correctly conclude that thermal release is negligible under Titan surface temperatures but becomes feasible at warmer layers. This is a meaningful improvement. However, they still do not include even a simple numerical order-of-magnitude estimate for non-thermal release pathways such as CR-UV photodesorption, solar-wind or GCR processing, or chemical kick-out. As a result, the plausibility of net HNC export from cold grains remains only qualitatively supported."

> *Authors reply:* Thank you. In response, we have added the following paragraph to the section entitled "Speculation on Astrochemical Relevance":
>
> "Once formed, HNC can remain bound to the surface or be released into the gas phase, either thermally when the particles are lofted into warmer atmospheric layers, or when they are exposed to UV photons or charged particle irradiation. While our Gibbs energy estimates suggest that temperatures around 180 K suffice for quantitative desorption on timescales of days, we speculate that non-thermal processes may accelerate such release and become dominant at lower temperatures. Galactic cosmic rays and their secondary particles also penetrates Titan's dense atmosphere, providing an additional energy source that can further process aerosol and ice surfaces over long timescales.[92] Modeling such processes lies beyond the scope of our work, but we can provide a simple estimate: solar UV photons with wavelengths greater than 220 nm penetrate deep into Titan's atmosphere, with photon fluxes of ~10^{10} and ~10^{7} photons cm^{-2} nm^{1} s^{-1} at ~200 and ~75 km, respectively.[29] Given typical values reported for molecular ices,[108–111] we adopt a representative photodesorption yield is 10^{-3} molecules per incident photon. If we further assume an effective spectral bandwidth of 50 nm, a polar surface exposure of 7%, and a 10% HNC surface coverage, we obtain desorption rates (per unit of total HCN crystal surface area) of order ~10^{6} and ~10^{3} molecules cm^{-2} s^{-1} at ~200 and ~75 km, respectively. These estimates suggests that UV-induced desorption pathways may occur on timescales of years at ~200 km, but not closer to the surface. Following HNC desorption, new HNC is regenerated that could either become trapped in the lattice or continue the release. This process would provide a dynamic route for modulating the gas-phase nitrile composition without requiring bulk sublimation of HCN ice. It could also contribute to the vertical redistribution and chemical evolution of nitriles in Titan's stratified atmosphere.

In the case of comets, observations have consistently shown that the gas-phase HNC/HCN ratio increases as comets approach the Sun.[65,71–75] This has been interpreted as evidence of in situ chemical production of HNC, rather than it being a parent molecule.[6,70,112] Our computed binding energy for HNC on HCN crystal surfaces (~75 kJ/mol) suggests that at low temperatures, HNC formed via surface-catalyzed isomerization would remain chemisorbed. However, near perihelion, solar heating can supply sufficient energy to desorb HNC from the outermost surface layer and release it into the coma. In addition to thermal desorption, non-thermal processes such as UV photodesorption and solar-wind sputtering should also be enhanced near perihelion. In this scenario, HCN crystals, if present, could act as catalytic reservoirs: storing HCN in the solid phase, converting a fraction into HNC at their polar surfaces, whilst releasing and producing more HNC when heated. Such processes align with observations of increased HNC production in comets at small heliocentric distances, where thermal and radiative processing of near-surface ices is most intense.[113]"

R3: "(2) Hybrid functional robustness check. The revised methods are much clearer overall, but the requested single hybrid-functional spot check under field for the key step is still missing. This should be added to bound self-interaction error and confirm that the field trends are not artefacts of the chosen GGA."

Authors reply: Thank you. The estimates we provide for this step (SI Figure S10) are not from GGA-level DFT but are DLPNO-CCSD(T)-refined energies on top of a structure optimized with hybrid-XC DFT, at the B3LYP-D3(BJ) level. We have clarified this further in SI Section 3.4:

"In our proposed HCN isomerization mechanism catalyzed by surface ionization, the removal of a terminal HNC molecule represents the rate determining step. To evaluate the feasibility of this process, we computed the DLPNO-CCSD(T)-refined gas-phase dissociation energies (ΔG) of HNC and, for comparison, HCN from a linear HCN chain in its neutral, protonated, or deprotonated form (Figures S10)."

R3: "(3) Astrochemical bridge. The new paragraph linking the mechanism to Titan and comets is well written and strengthens the relevance. Yet it remains a narrative argument. A short scaling or boxmodel estimate, even three or four lines, would greatly improve the quantitative credibility of the proposed contribution to observed HCN/HNC behaviour."

Authors reply: Thank you. We believe the approximate estimates we provide above addresses this comment.

R3: "(4) Language and scope. The manuscript continues to use strong "drive/catalyse" framing without the quantitative rate enhancement needed to fully justify it. In addition, the term "cosmic

wind" is non-standard. It should be replaced with precise terminology such as "solar-wind ions" for Titan and comets, or "galactic cosmic rays and secondary electrons" for dense-cloud environments. Unless the authors add quantitative rate support, the strongest claims in the title and abstract should be softened to "field-assisted pathways that may contribute under relevant conditions."

Authors reply: Thank you. Our calculations demonstrate that, once desorption is active (e.g., at elevated temperatures or under UV/particle irradiation), the surface mechanisms can in principle supply HNC on timescales that are compatible with observed behavior. In this sense, the mechanisms do represent a genuine rate enhancement relative to uncatalyzed gas-phase isomerization at low temperatures, even though we do not attempt to extract precise global rate coefficients. We have nevertheless modified the title slightly to say "Assist" instead of "Drive".

Our abstract now ends with the sentence:

> "Such field-assisted surface mechanisms may contribute to HCN-to-HNC isomerization under relevant conditions, and are suggested to explain part of the out-of-equilibrium abundance of HNC in cold environments such as Titan's atmosphere, and, potentially, in cometary comae."

We have also revised the caption of Figure 5 caption to use more precise terminology:

> "Solar wind and galactic cosmic rays are a well-known source of protons."
